# Critical thresholds of long-pressure reactivity index and impact of intracranial pressure monitoring methods in traumatic brain injury

**DOI:** 10.1186/s13054-024-05042-7

**Published:** 2024-07-29

**Authors:** Erik Hong, Logan Froese, Emeli Pontén, Alexander Fletcher-Sandersjöö, Charles Tatter, Emma Hammarlund, Cecilia A. I. Åkerlund, Jonathan Tjerkaski, Peter Alpkvist, Jiri Bartek, Rahul Raj, Caroline Lindblad, David W. Nelson, Frederick A. Zeiler, Eric P. Thelin

**Affiliations:** 1 Department of Clinical Neuroscience, Karolinska Institutet, Stockholm, Sweden; 2 Department of Neurosurgery, Karolinska University Hospital, Stockholm, Sweden; 3 Biomedical Engineering, Faculty of Engineering, University of Manitoba, Winnipeg, MB Canada; 4 Department of Molecular Medicine and Surgery (MMK), Karolinska Institutet, Stockholm, Sweden; 5 Department of Neurosurgery, Skåne University Hospital, Lund, Sweden; 6 Department of Radiology, Södersjukhuset, Stockholm, Sweden; 7 Department of Perioperative Medicine and Intensive Care, Karolinska University Hospital, Stockholm, Sweden; 8 Section of Perioperative Medicine and Intensive Care, Department of Physiology and Pharmacology, Karolinska Institutet, Stockholm, Sweden; 9 Department of Cardiology, Danderyd’s Hospital, Stockholm, Sweden; 10 Department of Neurosurgery, University of Helsinki, Helsinki, Finland; 11 Department of Neurosurgery, Uppsala University Hospital, Uppsala, Sweden; 12 Department of Medical Sciences, Uppsala University, Uppsala, Sweden; 13 Section of Neurosurgery, Department of Surgery, Rady Faculty of Health Sciences, University of Manitoba, Winnipeg, MB Canada; 14 Pan Am Clinic Foundation, Winnipeg, MB Canada; 15 Centre on Aging, University of Manitoba, Winnipeg, Canada; 16 Department of Neurology, Karolinska University Hospital, Stockholm, Sweden

**Keywords:** Pressure reactivity index, Traumatic brain injury, Neuro-monitoring, Intracranial pressure, Functional outcome

## Abstract

**Background:**

Moderate-to-severe traumatic brain injury (TBI) has a global mortality rate of about 30%, resulting in acquired life-long disabilities in many survivors. To potentially improve outcomes in this TBI population, the management of secondary injuries, particularly the failure of cerebrovascular reactivity (assessed via the pressure reactivity index; PRx, a correlation between intracranial pressure (ICP) and mean arterial blood pressure (MAP)), has gained interest in the field. However, derivation of PRx requires high-resolution data and expensive technological solutions, as calculations use a short time-window, which has resulted in it being used in only a handful of centers worldwide. As a solution to this, low resolution (longer time-windows) PRx has been suggested, known as Long-PRx or LPRx. Though LPRx has been proposed little is known about the best methodology to derive this measure, with different thresholds and time-windows proposed. Furthermore, the impact of ICP monitoring on cerebrovascular reactivity measures is poorly understood. Hence, this observational study establishes critical thresholds of LPRx associated with long-term functional outcome, comparing different time-windows for calculating LPRx as well as evaluating LPRx determined through external ventricular drains (EVD) vs intraparenchymal pressure device (IPD) ICP monitoring.

**Methods:**

The study included a total of n = 435 TBI patients from the Karolinska University Hospital. Patients were dichotomized into alive vs. dead and favorable vs. unfavorable outcomes based on 1-year Glasgow Outcome Scale (GOS). Pearson’s chi-square values were computed for incrementally increasing LPRx or ICP thresholds against outcome. The thresholds that generated the greatest chi-squared value for each LPRx or ICP parameter had the highest outcome discriminatory capacity. This methodology was also completed for the segmentation of the population based on EVD, IPD, and time of data recorded in hospital stay.

**Results:**

LPRx calculated with 10–120-min windows behaved similarly, with maximal chi-square values ranging at around a LPRx of 0.25–0.35, for both survival and favorable outcome. When investigating the temporal relations of LPRx derived thresholds, the first 4 days appeared to be the most associated with outcomes. The segmentation of the data based on intracranial monitoring found limited differences between EVD and IPD, with similar LPRx values around 0.3.

**Conclusion:**

Our work suggests that the underlying prognostic factors causing impairment in cerebrovascular reactivity can, to some degree, be detected using lower resolution PRx metrics (similar found thresholding values) with LPRx found clinically using as low as 10 min-by-minute samples of MAP and ICP. Furthermore, EVD derived LPRx with intermittent cerebrospinal fluid draining, seems to present similar outcome capacity as IPD. This low-resolution low sample LPRx method appears to be an adequate substitute for the clinical prognostic value of PRx and may be implemented independent of ICP monitoring method when PRx is not feasible, though further research is warranted.

**Supplementary Information:**

The online version contains supplementary material available at 10.1186/s13054-024-05042-7.

## Introduction

Moderate-to-severe traumatic brain injury (TBI) is a deleterious condition with a global mortality rate of about 30%, resulting in acquired life-long disabilities in many survivors [1]. Specialized neuro-critical care units (NCCU), where invasive monitoring is employed, have been shown to improve outcomes as compared to treatment in conventional critical care units [2, 3]. However, despite improvements in monitoring, about 40% of severe TBI patients deteriorate, presumably due to secondary brain injuries caused by a deranged metabolism, inadequate perfusion, and other intracranial insults [4, 5]. In our regional TBI database, we have seen 39% of patients present with secondary lesions, not seen on admission imaging, that are predominantly lesions of an ischemic nature [6]. Thus, better monitoring is required to improve outcomes and prevent potentially irreversible secondary cerebral injuries in severe TBI patients.

The pressure reactivity index (PRx), as a surrogate for cerebrovascular reactivity, has been suggested as a metric that could be monitored in order to prevent secondary insults such as pressure-passive ischemia or hyperemia by taking the intracranial auto-regulatory capacity into consideration [7]. PRx is commonly calculated by a moving Pearson’s correlation between intracranial blood pressure (ICP) and mean arterial pressure (MAP), averaged over a 10-s period, using 5-min moving time-windows [7–10]. PRx ranges from − 1 (intact autoregulatory capacity) to 1 (impaired autoregulatory capacity), with established critical thresholds of PRx > 0.35 and > 0.25, and > 0.05 being associated with mortality and unfavorable outcomes at 6 months, respectively [11–15].

However, the problem with PRx is that it requires high-resolution data and potentially expensive information technology (IT)-solutions, which has resulted in it being used clinically in only a handful of centers worldwide. As a solution to this, a low resolution PRx has been suggested, known as Long-PRx or LPRx [16, 17]. Previous studies have looked at time-windows from 5 to 240 min and found that LPRx holds similar outcome predictive capacity as PRx [16, 18–21]. Yet, studies calculating both PRx and LPRx in the same cohort found PRx to have higher associations with outcomes than LPRx [22, 23]. However, we studied a smaller cohort analyzing down-sampled PRx and ICP/MAP values which indicated that while a lot of granularity in the data is missed, when going to minute-by-minute data for ICP/MAP with 20-min time windows for LPRx derivation, a similar time-domain statistical structure for PRx and LPRx exists [21, 24]. Thus, the vector-domain temporal relationships between ICP and MAP is preserved, providing confidence in the ability of LPRx to assess some aspects of cerebral autoregulation [24]. However, such work has been limited to date, and thus LPRx as a measure still remains underexplored.

As of today, there is only a single center cohort study that has investigated the critical thresholds of LPRx in TBI [21], furthermore several studies have used different cut-offs and time-windows [16, 18–23]. Thus, it is still unclear which time window and threshold of LPRx is most appropriate, or if existing published critical thresholds for standard PRx can be used for LPRx monitoring. Moreover, almost all previous studies combine ICP monitoring of intraparenchymal devices (IPD) and external ventricular drains (EVD). ICP worldwide is still measured using EVD (while only 15% in Europe, EVDs are believed to constitute a majority in low-and-middle-income-countries (LMIC)) [25–27], making it important to establish a method that works for both types of acquired ICP.

Hence, this observational study aims to explore LPRx within a large TBI database to A) establish critical thresholds of LPRx that are associated with long-term functional outcome, B) determine which time-window for calculating LPRx is optimal for outcome prediction, and C) investigate if LPRx derived from EVD differs from intraparenchymal ICP devices. Our hypothesis is that similar thresholds as seen for PRx will be valid for LPRx, and that time-windows up to 20 min will be similar as 5-min time windows.

## Materials and methods

### Study design

From between January 1, 2006 to December 31, 2019 patients admitted to the adult NCCU at Karolinska University Hospital, Stockholm, Sweden, a level one trauma center, with moderate and severe TBI (diagnosed as Glasgow coma scale (GCS) <  = 8 and > 15 years old) were included in this study. All patients had invasive ICP and MAP monitoring for more than 6 h that was archived in high-frequency (1–5-min median levels) and were retrospectively analyzed (n = 435) in this observational study. Treatment was mediated according to local guidelines in general concordance to that of the Brain Trauma Foundation (BTF) [2, 28, 29], and is described in detail elsewhere [30]. These patients were mechanically ventilated, with arterial partial pressure of CO_2_ (PaCO_2_) targets used, where normal to mild hyperventilation (defined here as PaCO_2_ 4.5–5 kPa) was commonly applied as one of several to manage increased ICP. Head of the bed was commonly elevated 30 degrees and cerebral perfusion pressure (CPP) was calculated with the arterial pressure transducer placed at the level of the tragus (some patients had a dual transducer to measure arterial blood pressure both at the cardiac and cerebral level) [31]. As part of our local patient registry, Glasgow Outcome Scale (GOS) was prospectively acquired through questionnaires and telephone interviews at about 12 months following injury [32].

### IRB ethics

This study was approved by the Swedish Ethical Review Authority (#2020–05227) on November 17, 2020 and adheres to the Helsinki Declaration of 1975.

### Data collection

The patient data collection was identical to that previously described [33]. As a summary, all patient demographics, injury and treatment information were either manually collected by a medical professional from the electronic hospital chart system Take Care (CompuGroup Medical Sweden AB, Stockholm, Sweden) or automatically recorded using Clinisoft (Centricity Critical Care, CCC, General Electric Company, Boston, USA). The worst pre-sedation/intubation GCS score was used. Pre-hospital hypoxia (oxygen saturation < 90%) or hypotension (systolic blood pressure < 90 mmHg) were registered from the scene of the accident, or at the hospital admission [3]. The admission computerized tomography (CT) scan was assessed using the Marshall CT classification [34]. Primary decompressive craniectomy was defined as a craniectomy performed as initial surgery (i.e. where the bone flap was not returned following initial evacuation surgery or due to diffuse injury and brain swelling), while a secondary decompressive craniectomy was defined as a hemicraniectomy performed at least 48 h after trauma due to refractory high ICP [35].

MAP was obtained through either radial or femoral arterial lines connected to pressure transducers (Baxter Healthcare Corp. CardioVascular Group, Irvine, CA, or similar devices). ICP was acquired via an intra-parenchymal strain gauge probe (Codman ICP MicroSensor; Codman & Shurtleff Inc., Raynham, MA, USA), raumedic catheter Neurovent-P (Raumedic AG, Münchberg, Germany), parenchymal fiber optic pressure sensor (Camino ICP Monitor, Integra Life Sciences, Plainsboro, NJ, USA; https://www.integralife.com/) or using EVD (Medtronic, Minneapolis, MN, USA). Both the MAP and ICP data were clean from data artifacts by using direct visual inspection and threshold limits (0 < ICP < 80 mmHg and 0 < MAP < 400 mmHg). ICP data when drains were opened were identified by manual indications in CCC, verified by manual inspection, and were removed. Thus, all time that EVD had open cerebral spinal fluid drain was removed.

### Signal processing

Data collected was stored in the database as the median for each time period, predominantly that of 2 min, however ranging from 0.5 to 5 min (1 min median, interquartile rate of 1–2 min), generating unevenly sampled time series data (Appendix A for more details). It should be noted that CCC was not designed as a research tool, thus the reason behind why ICP and MAP values were sampled irregularly is hard to fully identify. Though some reasons include, data recording policy, adjustments in storage allotments, and modified sampling rate of the CCC system over the years. We performed two complete analyses on this database including one which we imputed the data to give regularly sampled data and one which used the data as is (with the sporadic sampling). Further details on the imputation method can be found in Appendix B, though given that the overall results were nearly identical (statistically similar for all key thresholds), the sporadic data (non-imputed) will be demonstrated and referenced for the rest of this manuscript. For all tables, the data is represented as grand means for each patient summed with median levels and interquartile ranges.

From the values of ICP and MAP, low-frequency PRx (LPRx) was derived via the moving Pearson’s correlation coefficient of multiple consecutive minute-by-minute samples, and calculated every minute [18, 20, 36]. LPRx values range from − 1 to 1, with higher values indicating increasingly impaired cerebrovascular reactivity as indicated by slow fluctuation responses of ICP to MAP changes. LPRx was calculated using 10, 15, 20, 30, 60, 90 and 120 consecutive samples (10–120 min of time) and labeled as: LPRx_10, LPRx_15, LPRx_20, LPRx_30, LPRx_60, LPRx_90 and LPRx_120; in line with previous literature on LPRx in TBI [18, 20, 36].

### Statistical analysis

All statistical analysis was performed using R statistical computing software (R Foundation for Statistical Computing (2020), Vienna, Austria, http://www.R-project.org/). This manuscript performed an exploration into the relationships between final GOS (last registered GOS) and various overall mean cerebral/physiological responses. From this data the overall mean values for LPRx and ICP were calculated for the entire patient recording, the first 24/48/96/144 h (1/2/4/6 days) and daily times (days 1–7).

Pearson’s chi square test was used to find the best threshold for ICP and LPRx values in analogues with past work [11, 37]. The data was dichotomized by different thresholds, above/below thresholds from − 0.5 to 0.7 (with incremental steps of 0.05) for LPRx and 0 to 40 (with incremental steps of 0.5 mmHg) for ICP. Chi-squared tests were then performed between each dichotomized threshold and outcome. Outcomes were defined as survival (GOS 1 vs. 2–5) [38] or favorable outcomes (GOS 1–3 vs. 4–5) [32]. For each threshold a chi-square statistic was calculated. The threshold with the highest chi-square statistic was assumed to have the best discriminative value for outcome, indicating that this threshold value had the most accurate categorization of the patient population. This procedure was repeated for all time periods (mean values of the full monitoring time, first 1/2/4/6 days and each of the first 7 days) as well as after creating subgroups according to EVD vs IPD. We also performed chi-squared analysis on patients without a decompressive craniectomy.

Next using the same chi-squared technique as previously described, the method was repeated for % time LPRx over key threshold (> 0, > 0.2 and > 0.3). These thresholds were chosen based on previously defined PRx thresholds (which are similar to the ones found in this manuscript) [12, 23, 37, 39]. Again, the threshold with the highest chi-square statistic was assumed to have the best discriminative value for outcome, indicating that this % time LPRx above the threshold value had the most accurate categorization of the patient population. This procedure was repeated for all time periods (% time LPRx above threshold for the full monitoring time, first 1/2/4/6 days and each of the first 7 days) as well as after creating subgroups according to EVD vs IPD.

Basic physiological statistic of each of MAP, ICP and LPRx was compared using a Mann-U test of their overall distribution for the survival and favorable outcomes groups. P values were not adjusted for multiple comparisons, with overall alpha of significance set to 0.05.

## Results

### Patient characteristics

**Fig. 1:**
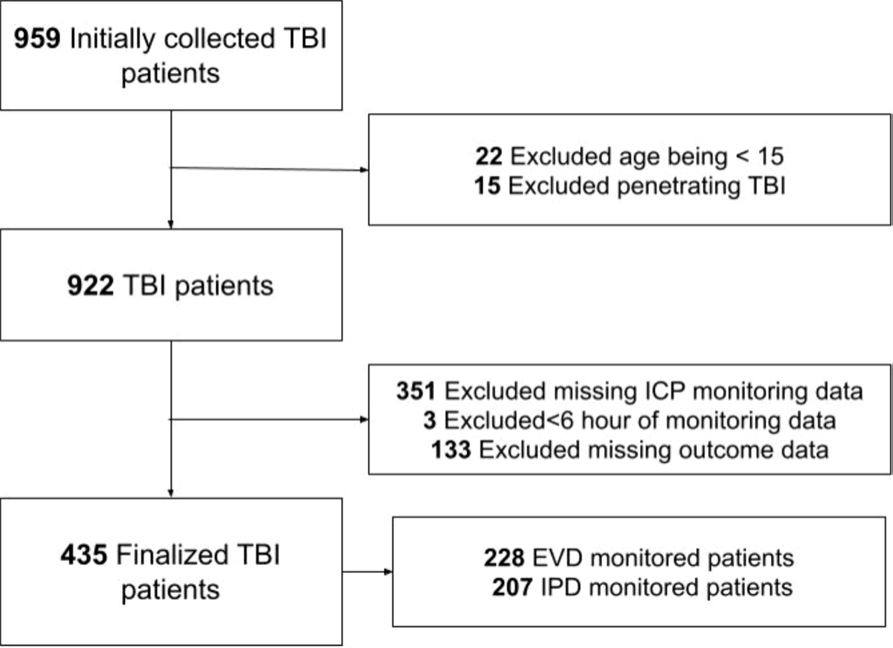
Patient Selection The selection of the patient data from Stockholm, with inadequate monitoring demonstrating limited physiological data(< 6 h) or missing data for key physiologies. The remaining n = 435 patients represent ICU TBI patients requiring invasive monitoring to optimize recovery. EVD, external ventricular drain; IPD, intraparenchymal monitor; TBI, traumatic brain injury

N = 435 patients were eligible for the final analysis (Fig. 1), of whom 207 had IPD and 228 had EVD. One patient had both monitors placed, for whom only EVD data was used for analysis. The median age was 51 years (interquartile range; IQR: 33–62.5 years), with 338 (77.7%) being males (Table 1). 277 patients had at least 6 days of recorded physiology and 432 have at least a full day of recording. It should be noted here that the artifact removal resulted in, on average, less than 1% of the data loss per patient, however for some patients (mostly EVD drainage patients) this was up to 40% of the time (though this was rare, in 10 patients). In total, 260 (59.8%) had intracranial mass lesions removed, and 44 (10.1%) has either a primary- or secondary decompressive hemicraniectomy. TBI demographics are in keeping with normal TBI cohorts. Appendix C describes admission characteristics and type of monitoring for each year of recording, including outcome.

**Table 1:** Mann–Whitney U/Chi-Square Analysis of Physiologic and Demographic Data for Alive vs Dead and Favorable vs Unfavorable

Variable	Alive/dead outcome groups			Favorable/unfavorable outcome groups		
	Dead median (IQR)/ number (%)	Alive median (IQR)/ number (%)	p value	Unfavorable Median (IQR)/ Number (%)	Favorable Median (IQR)/ Number (%)	p value
Number of patients	84 (19%)	351 (81%)		221 (51%)	214 (49%)	
Age (years)	59.5 (44–69)	49 (30–61)	**p < 0.0001**	58 (44–68)	44.5 (28.2–57)	**p < 0.0001**
GCS eye	1 (1–1)	1 (1–2)	0.0185	1 (1–2)	1 (1–3)	**0.0058**
GCS motor	2 (1–4)	4 (2–5)	**p < 0.0001**	3 (1–5)	5 (3–5)	**p < 0.0001**
GCS Verbal	1 (1–1)	1 (1–2)	**0.0026**	1 (1–2)	1 (1–3)	**0.0011**
GCS	5 (3–7.5)	7 (5–10)	**p < 0.001**	6 (4–8)	7 (5–10.5)	**p < 0.0001**
Pupils Bilat Unreactive	33 (39.3%)	52 (14.8%)	**p < 0.0001**	58 (26.2%)	27 (12.6%)	**p < 0.001**
Pupils Unilateral Unreactive	11 (13.1%)	50 (14.2%)	0.786	33 (14.9%)	28 (13.1%)	0.5798
Pupils Bilat Reactive	50 (59.5%)	277 (78.9%)	**p < 0.001**	146 (66.1%)	181 (84.6%)	**p < 0.0001**
Sex (Male)	68 (81%)	270 (76.9%)	0.4264	171 (77.4%)	167 (78%)	0.8689
Hypoxia	36 (42.9%)	99 (28.2%)	**0.0092**	85 (38.5%)	50 (23.4%)	**p < 0.001**
Hypotension	31 (36.9%)	110 (31.3%)	0.3284	81 (36.7%)	60 (28%)	0.0553
*Marshall CT Score*						
V-VI	50 (59.5%)	183 (52.1%)	0.2234	123 (55.7%)	110 (51.4%)	0.3746
IV	3 (3.57%)	13 (3.7%)	0.9551	8 (3.62%)	8 (3.74%)	0.9487
III	15 (17.9%)	59 (16.8%)	0.8192	36 (16.3%)	38 (17.8%)	0.6846
II	16 (19%)	102 (29.1%)	0.0641	57 (25.8%)	61 (28.5%)	0.5254
I	0 (0%)	2 (0.57%)	0.5465	1 (0.452%)	1 (0.467%)	0.9844
Traumatic subarachnoid- or intraventricular hemorrhage	71 (84.5%)	287 (81.8%)	0.5529	190 (86%)	168 (78.5%)	0.0416
Epidural Hematoma	4 (4.76%)	64 (18.2%)	**0.0023**	16 (7.24%)	52 (24.3%)	**p < 0.0001**
Surgical evacuation of lesions	50 (59.5%)	210 (59.8%)	0.9596	129 (58.4%)	131 (61.2%)	0.5461
Decompressive Craniectomy Primary	13 (15.5%)	22 (6.27%)	**0.0054**	24 (10.9%)	11 (5.14%)	**0.0286**
Decompressive Craniectomy Secondary	3 (3.57%)	6 (1.71%)	0.2829	8 (3.62%)	1 (0.467%)	**0.0212**
ICU Length of Stay (Days)	5.9 (2.91–13.6)	11 (5.5–16)	**p < 0.001**	11 (4.5–16.4)	10 (5–15.5)	0.627
MAP (mmHg)	76.8 (74.2–81.3)	78.3 (74.2–82.6)	0.3332	77.5 (74.2–82.2)	78.6 (74.3–82.7)	0.2685
ICP (mmHg)	15.8 (12–31)	11.1 (8.43–13.6)	**p < 0.0001**	12 (8.68–15.7)	11.5 (8.9–13.9)	**0.0355**
CPP (mmHg)	60 (47.6–65.6)	67 (62.6–71.6)	**p < 0.0001**	65.1 (60–70.2)	66.9 (62.4–71.5)	**0.001**
LPRx_10 (au)	0.226 (0.0617–0.416)	0.0282 (-0.0744–0.127)	**p < 0.0001**	0.0892 (-0.033–0.226)	0.0208 (-0.0817–0.114)	**p < 0.0001**
LPRx_15 (au)	0.231 (0.0559–0.391)	0.0269 (-0.0842–0.127)	**p < 0.0001**	0.0798 (-0.035–0.242)	0.0306 (-0.0878–0.11)	**p < 0.0001**
LPRx_20 (au)	0.207 (0.0544–0.396)	0.0245 (-0.0803–0.128)	**p < 0.0001**	0.0797 (-0.0331–0.243)	0.0207 (-0.0907–0.109)	**p < 0.0001**
LPRx_30 (au)	0.241 (0.0613–0.395)	0.0273 (-0.0799–0.144)	**p < 0.0001**	0.0825 (-0.0386–0.259)	0.0243 (-0.0852–0.128)	**p < 0.0001**
LPRx_60 (au)	0.221 (0.0596–0.385)	0.0414 (-0.0592–0.161)	**p < 0.0001**	0.102 (-0.0259–0.266)	0.0369 (-0.0738–0.147)	**p < 0.001**
LPRx_90 (au)	0.173 (0.0663–0.392)	0.0624 (-0.0385–0.168)	**p < 0.0001**	0.112 (-0.00988–0.266)	0.0615 (-0.0435–0.162)	**p < 0.001**
LPRx_120 (au)	0.186 (0.0748–0.39)	0.0697 (-0.0235–0.186)	**p < 0.0001**	0.118 (0.00725–0.275)	0.0744 (-0.036–0.184)	**0.0013**

The full time for each patient recording was used to find the mean value for each indicated index, bold values indicate significance. Au = arbitrary units, CPP = cerebral perfusion pressure, CT = computed tomography, GCS = Glasgow Coma Scale, ICP = intracranial pressure, ICU = intensive care unit, IQR = interquartile range, LPRx = long pressure reactivity index, MAP = mean arterial pressure, mmHg = millimeters of mercury

### Critical thresholds for outcome prediction for LPRx

**Fig. 2:**
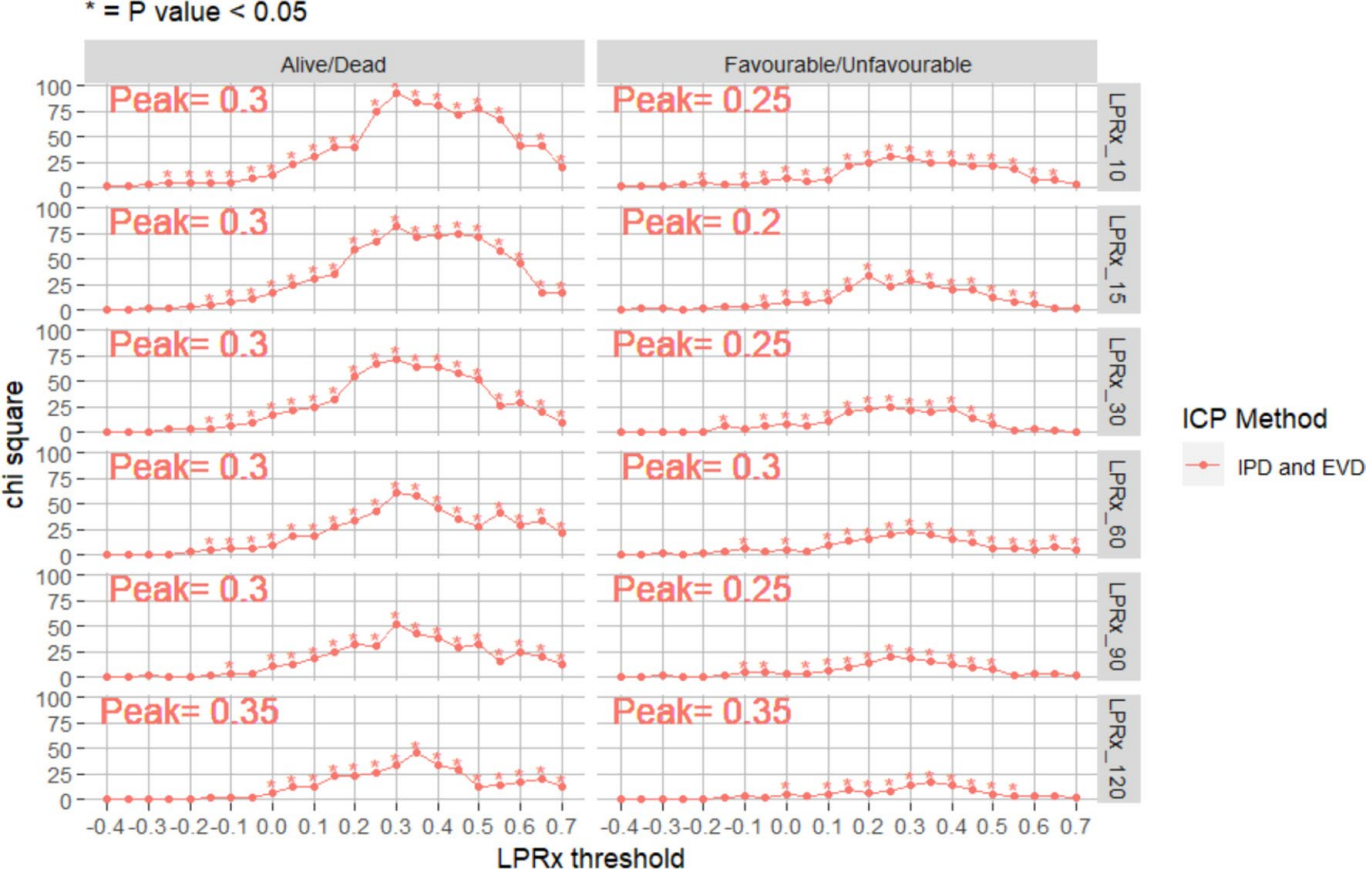
Outcome for LPRx The most optimal dichotomized threshold from all the LPRx values was encompassing 0.25–0.35, using both EVD and IPD monitoring for the full time of data (whole measurement period for all patients). EVD, external ventricular drain; IPD, intraparenchymal monitor; LPRx, long pressure reactivity

The sequential chi-square method was performed for each LPRx window (LPRx_10/LPRx_15/LPRx_20/LPRx_30/LPRx_60/LPRx_90/LPRx_120, i.e. 10 to 120 min window derived correlation coefficients). Plots presenting the chi-square values for incremental thresholds of mean LPRx found over the full recording of each patient was completed for each parameter, both Alive vs Dead and Favorable vs Unfavorable, presented in Fig. 2. For each plot, the threshold resulting in the highest chi-squared value was identified as the critical threshold.

For most of the cerebrovascular reactivity indices, similar critical thresholds were found for Alive vs Dead and Favorable vs Unfavorable outcome. The LPRx_10 and LPRx_15 plots produced peaks at 0.25 or 0.3 for both outcome types, though the Alive vs Dead dichotomization had improved chi-squared values compared to Favorable vs Unfavorable outcome. The longer periods of time (LPRx_30, LPRx_90, and LPRx_120) had a slightly higher critical threshold of 0.3 or 0.35 and a strong chi-squared for the Alive vs Dead categorization. This is in alignment with the findings tabulated in Table 1 and the Appendices.

Finally, the longer the LPRx time window the lower overall chi-squared value, with the 10 min window having the most significant chi-squared value.

There was a limited impact of monitoring time on LPRx thresholds, with thresholds varying between 0.2–0.35 for durations of monitoring from 1 to 6 days (Appendix D/E). However, when investigating critical LPRx thresholds based solely on individual daily mean values (0–24/24–48/48–72… hours) chi-squared values were notably lower and a decrease in the threshold was seen as the recording was further from the initial time of care (Appendix F/G with Appendix H summarizes the daily patient demographics). This is notable in the 4th to 5th day of recording, with almost all significance of LPRx lost after the 5th day (lower overall chi-squared and increased p values).

### Critical thresholds of LPRx – Impact of ICP Monitoring Method

**Fig. 3:**
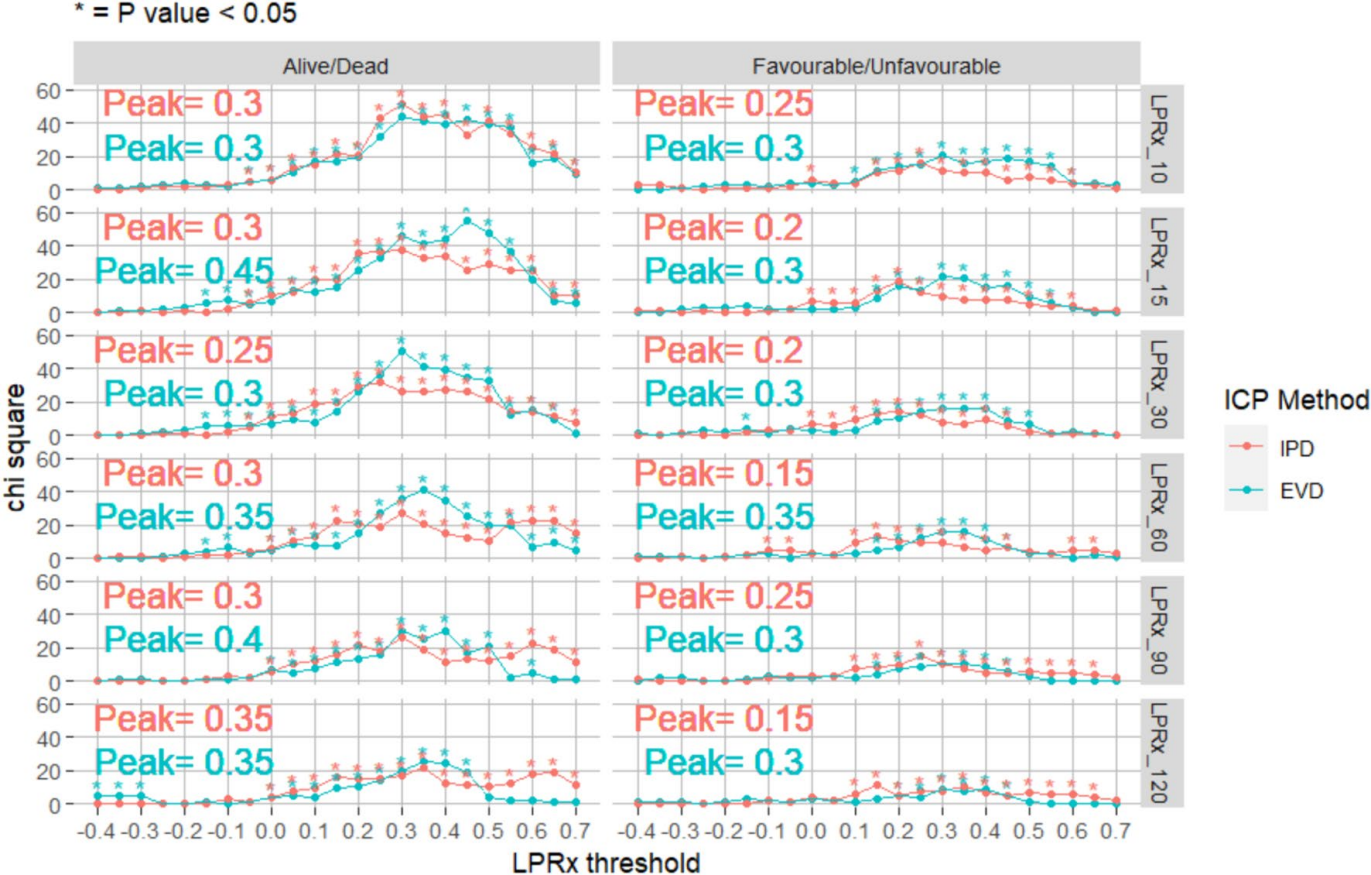
LPRx for Different ICP Monitoring Methods The figure displays different LPRx windows and the resulting thresholds with different ICP monitoring methods. Similar overall results with IPD and EVD. Noting also that as LPRx increase in time the chi-squared values decrease. EVD, external ventricular drain; ICP, intracranial pressure; IPD, intraparenchymal monitoring; LPRx, long pressure reactivity; _10, 10 min window; _15, 15 min; _30, 30 min; _60, 60 min; _90, 90 min; _120, 120 min

The sequential chi-square method was performed for each ICP monitoring method; EVD, IPD and these combined into one group. Figure 2 demonstrates EVD and IPD in one group and Fig. 3 demonstrates just EVD and just ICP for the full time (Appendix I/J shows patient demographics for IPD and EVD). Overall, there was a similar response between EVD and IPD monitoring of ICP and derived LPRx measures (both overall mean values and found thresholds), with peak values at 0.2–0.35 thresholds. There was limited difference seen in the patients without a decompressive craniectomy (Appendix P).

### Critical thresholds for outcome prediction for ICP

The sequential chi-square method was performed for ICP, for patients with both EVD + IPD, just EVD and just IPD (Appendix K). As indicated in Appendix K, the EVD-based critical threshold for mean ICP was lower (16.5 mmHg) than IPD-based critical threshold at (20/24 mmHg).

Appendix K/L/M, show the time in NCCU and the overall impact of the ICP critical thresholds. To note, the first 2 days of care appeared to have the most significance, with ICP data after the 4th day losing a significant amount of its discriminative capacity (as reflected in a reduced overall chi-squared magnitude).

### % Time of LPRx above critical threshold

All analysis for this aspect can be found in Appendices Q-Y, which document the association between % time LPRx was above each key threshold (> 0, > 0.2 and > 0.3) and outcome. Overall, for % time LPRx > 0.3 about 50% of the time was an indicator of poor outcome. For % time LPRx > 0 was about 70–80% of the time, which corresponds with what would be expected. Around the 3/4th day, the overall discriminative capacity of LPRx decreased, with the higher LPRx calculated windows (LPRx_60 to LPRx_120) having lower overall peak chi-squared values.

## Discussion

This is the first manuscript to extensively explore and derive key critical thresholds for outcomes association for LPRx over various time windows. Proceeded by recent work from Riemann where three time windows were explored [21], this current study offers unique insights into this surrogate measure of cerebral autoregulation. This preliminary work has confirmatory findings on LPRx which indicates that it has similar overall prognostic thresholds as standard PRx, thus as a clinical measure LPRx is likely a substitute for PRx. Our results confirmed similar LPRx thresholds between IPDs and closed EVDs, though external validation of our results will be important.

As this was the second study to investigate critical thresholds for LPRx in a large TBI population, it bears highlighting that a LPRx calculated over a 10 to 120-min window (with low 1–2 min samples) displayed thresholds of 0.2–0.35, similar to that of PRx (that uses 10 s samples) [11, 12, 14, 21]. Though Riemann’s past work on LPRx also saw a significant drop in chi-squared magnitude for larger LPRx calculation windows, they found LPRx to have highly variable critical thresholds and overall a lack of statistical significance (for more information see Table 2) [21]. Therefore it is likely that LPRx windows capture many prognostic factors associated with PRx, with larger LPRx windows reducing its discriminative capacity. Furthermore, LPRx maybe a viable substitute for PRx clinical calculations in situations where PRx cannot be found, including artifact prone signals (monitoring effects from nurses) and provide a route to personalized cerebral autoregulation assessment in situations where the monitor data is diminished (low MAP/ICP yields or low minute plus, sampling rates). While this data comes from a low-resolution legacy system (CCC), we are not trying to promote a specific system but highlighting that low-resolution PRx is a viable surrogate in place where PRx can not be found, displaying similar historical chi-square thresholds [11, 12, 14, 21]. However, for prospective monitoring of cerebrovascular reactivity measures today, bedside systems with this data available are more suitable.

**Table 2:** Past Literature on Pressure Reactivity Calculations

Reference	Year	Sample size	Sample location	Sampling frequency of ICP/MAP	Method for pressure reactivity monitoring	Identified threshold
*ICP Dervied Autoregulation Index*						
Sorrentino et al. [11]	2012	459	Addenbrooke Hospital, Cambridge, UK	8 s	30 consecutive values	Optimal chi-squared 0.05 for favorable and 0.25 for survival outcome
Zeiler et al. [12]	2018	358	Addenbrooke Hospital, Cambridge, UK	10 s	30 consecutive values	PRx threshold of 0.35 for both favorable and survival (p < 0.001)
Riemann et al. [21]	2020	855	Addenbrooke Hospital, Cambridge, UK	10 and 60 s	30 and 20, 60, 240 consecutive values	PRx, the critical threshold for favorable 0.25 and 0.2 for survival LPRx_20 both favorable and survival of 0.05 LPRx_60 favorable of 0.45 and 0.15 for survival LPRx_240 favorable of 0.00 and 0.25 for survival (p value not indicated)
Stein et al. [14]	2023	345	CAnadian High-Resolution TBI (CAHR-TBI) Research Collaborative Canadian Hospitals	10 s	30 consecutive values	PRx two critical thresholds at 0.15 and 0.55 for survival prediction and 0.1 and 0.55 for favorable outcome prediction (p < 0.001 for all)
Hong et al. (current study)	2024	EVD = 237, IPD = 218 (total 455)	Karolinska University Hospital, Stockholm, Sweden	60 s	10 to 120 consecutive values	LPRx threshold of 0.15 to 0.25 (p < 0.001) using chi-squared testing, longer LPRx the higher the over LPRx threshold for both favorable and survival
*TCD Derived Autoregulation Index*						
Sorrentino et al. [37]	2011	763	Addenbrooke Hospital, Cambridge, UK	10 s	30 consecutive values	Mx_a threshold of 0.3 for both favorable (p = NS) and outcome (p = 0.003)
Zeiler et al. [40]	2017	281	Addenbrooke Hospital, Cambridge, UK	10 s	30 consecutive values	Sx_a displayed thresholds of -0.10 (p < 0.001) for favorable and 0.05 (p = 0.019) for survival Dx_a failed to display any statistically significant threshold
*Near Inferred Blood Oxygen Spectroscopy Derived Autoregulation Index*						
Gomez et al. [41]	2024	129	CAnadian High-Resolution TBI (CAHR-TBI) Research Collaborative Canadian Hospitals	10 s	30 consecutive values	COx and COx_a displayed thresholds of 0.2 (p < 0.001) for favorable and survival

COx, cerebral oximetry index (cerebral perfusion pressure and regional blood oxygen saturation); COx_a, cerebral oximetry index (MAP and regional blood oxygen saturation); Dx_a, diastolic pressure reactivity (diastolic cerebral flow velocity and MAP); EVD, external ventricular drain; ICP, intracranial pressure; IPD, intraparenchymal monitoring; LPRx, long pressure reactivity (minute by minute values of MAP and ICP; 20 to 240 consecutive values); MAP, mean arterial blood pressure; Mx_a, mean pressure reactivity (mean cerebral flow velocity and MAP); PRx, pressure reactivity (10 s values of MAP and ICP); Sx_a, systolic pressure reactivity (systolic cerebral flow velocity and MAP)

Furthermore, past work has shown that patients with long periods of time in a dysautoregulation state (PRx > 0–0.3) have overall worse outcomes [12, 15, 37, 39, 42, 43], reciprocated within our work. Therefore this highlights that like PRx, LPRx likely has similar descriptive information about outcomes as PRx. All of this is in line with past work discussing the lower limit of autoregulation (LLA), where LLA describes low systemic blood pressure linked with dysautoregulation [44–50]. LLA has been clearly documented in animal models, where systemic blood pressure was decreased [45, 49–52]. In some of this work the LLA was linked with a PRx value of ~ 0.3 [45, 49]. Such work has been expanded develop an individualized measure of care, with the optimal cerebral perfusion pressure (CPPopt) gaining extensive exploration in TBI care [36, 53, 54]. CPPopt uses the association between systemic blood pressure and PRx to provide a targetable personal value of care. Though this is still in its feasibility stage CPPopt, has demonstrated both prognostic and associations with outcome, with emerging work evaluating its impact [36, 54, 55]. All this work may benefit from LPRx as a substitution for PRx, where the more momentary assessment of PRx (10 s) is not feasible.

However, it should be noted that though we have demonstrated a prognostic similarity between LPRx and PRx, this does not indicate that these measures can be fully interchangeable. Many of the fast vasogenic aspects surrounding PRx determine calculation times, would likely be diminished at the larger time windows used by LPRx [56, 57]. This may account for the decrease in overall chi-squared values as the LPRx calculation window increases. Moreover the cerebrovascular reactivity factors at higher frequency ranges (< 1 min) would be impossible for LPRx to capture [56, 57]. Fundamentally current PRx/LPRx measures are derived from the correlated MAP and ICP values, and though factors that dramatically influence blood pressure likely influence PRx, recent work on decompressive craniectomies has demonstrated PRx had similar pathophysiological information pre/post treatment (reciprocated in our work) [58, 59].

With the current cerebrovascular reactivity measures, there is a limitation with the direct thresholding method used in this study, as there is a wide individual range of optimal patient thresholds (the range for significant values ranges from 0 to 0.5). Moreover, compared to the strong relationship with survival, favorable outcomes had lower overall chi-squared values and a less distinct peak (in keeping with past literature) [12–15, 60]. Presumably, the immediate deranged intracranial dynamics will play a smaller part in the long-term outcome prediction of survivors as compared to those that succumb from their injuries. Thus, though the dichotomization method for determining a threshold for a global population has some value, the more individual factors that drive LPRx in each patient needs to be explored (this is an issue for all cerebrovascular reactivity measures currently used) [11, 12, 24]. Again it should be re-emphasized that the post-hoc analyses generating chi-squares that we have performed are more for comparing our low-resolution PRx with that of other PRx papers and thus have directly replicated the thresholding analyses (which is what is current widely referenced and quoted PRx thresholds in the literature and clinical guidelines) [11, 12, 14, 21]. It must be mentioned that this method only provides a prognostic threshold, as the method of dichotomization focuses on long-term outcome scoring systems and thus does not necessarily represent a pure physiologic threshold, but an epidemiologic one. Though, pre-clinical literature does support some relation between cerebrovascular reactivity thresholds of ~  + 0.2–0.3 and identification of the LLA during systemic hypotension and intracranial hypertension, using both ICP and infrared based metrics [45, 49].

When analyzing the time and these individual LPRx measures, they appeared to sufficiently indicate similar overall values for critical thresholds associated with outcomes (ie. first 1–6 days had similar LPRx thresholds). However, when splitting up the data into each daily measure, from day one to day six, it was found that the dichotomization of the thresholding methodology lost its significance as the time got further from day one. This is likely due to a number of factors, though primarily the fact that extreme patients (either dead or fast recovery patients) would be removed from the data recording, focusing in on more dynamic patient cases as the time goes on. Moreover, given the fact that the longer a patient spends in the NCCU in theory would result in their overall intracranial physiology to move to normality, and the one-to-one thresholding for this time would be less indicative of an initial NCCU state as well as less responsive in overall physiological derangement.

Given the nature of this population, we had the unique opportunity to evaluate the two most common ICP monitoring methodologies, that being EVD and IPD methodologies. As EVD and IPD monitoring allows for different routes of care such as allowing cerebrospinal fluid (CSF) drainage, it makes populations with only EVD monitoring relevant to study. During periods of closed EVDs, we noticed that the LPRx measures performed similar regardless of monitoring device. This is in line with previous work showing that EVD and CSF drainage has a limited overall impact on the derived cerebrovascular reactivity index [61–63]. However, it should be noted that EVD patients had a slightly lower chi-squared value and lower overall LPRx/ICP critical thresholds, compared to those with LPRx derived from IPD devices, which may be explained by lower ICP values in the EVD group compared to IPD group. EVD drainage allows for a simplistic modification of brain pressure (particularly the lowering of overall ICP values, seen within this population with lower overall ICP thresholds observed). However, there was still a significant threshold seen with the EVD-based measures.

## Limitations

Despite the over 400 patients within this analyzed population, there are still significant limitations to overall heterogeneity and cofactor considerations. The segmentation of the data based on ICP monitoring method resulted in about 200 patients within each category. Although effective as overall gross mean assessments, the cohort has a lot of heterogeneity regarding TBI injury pattern, demographics and overall patient care, factors not accounted for within this analysis. To evaluate the effects of these potential confounders, a larger patient cohort would be needed.

Although this study uses similar methodology as in earlier studies, it has its limitations. Particularly in the fact that the more individualized momentary physiological aspects associated with patient care are not accounted for. As time and care grow the direct response of these impaired states would in theory be mitigated or at least minimized and thus the noted associations from extreme cases (ie, the first days) would likely not be seen in the later days as seen within this population. To address this, more momentary assessment and personalized evaluation of physiological treatment should be completed.

Past work exploratory work on PRx use has used the chi squared approach to approximate the threshold that has the best discriminative capacity with the data. Though useful to explore the data, such a technique is favoring the best bifurcation of the data and does not account for potentially more relevant clinical factors (like what threshold is the patient in danger or outlier patient who may have higher overall risk). Thus, for future work defining clinical thresholds for variables, other methods need to be explored. Methods that use an area under curve that focuses on preserving sensitivity well maximizing specificity provides more clinically relevant information. This is because it is less prone to withdraw needed treatment and favors assessing cases where the patient may be in danger. Therefore, when implementing LPRx/PRx in larger data modeling, exploring the optimal threshold through the diagnostic accuracy approach would be of benefit.

Next, PRx as a method of cerebrovascular reactivity determination is less robust than new methods like that of the pulse amplitude index or wavelet PRx [39, 64–66]. However, LPRx within this population appeared to have sufficient accuracy as to drive sufficiently similar PRx critical threshold values thus when data limitations exist, LPRx method can be considered.

Finally, as per the retrospective nature of this study, it is likely that some patients were withheld treatment due to severe injuries which are not deemed survivable, or per the known wishes of the patient or those of the next-of-kin. This is difficult to fully adjust for, but treatment withdrawal is generally uncommon at our institution. Likewise, we know from previous experience with the same cohort that few of the in-hospital mortality cases were due to withdrawal of treatment for the TBI itself and likely due to multi-organ failure [67].

## Conclusion

For LPRx determined over 10 to 20-min windows we found a critical threshold of 0.25, which is similar to past studies using PRx thresholding values, indicating that our LPRx has similar clinical prognostic value as PRx. Therefore, in a clinical setting where high frequency PRx cannot be determined, LPRx is likely a sufficient substitute. As LPRx is found using only minute-by-minute samples of MAP and ICP (with as low as 10 samples), it therefore opens the use of LPRx in more clinical centers globally. Next, as EVD and IPD derived LPRx performed similarly, it indicates that despite the intermittent CSF draining, LPRx can still be clinically determined. Therefore as a clinical prognostic measure LPRx is an adequate substitute for PRx, though more research is warranted to study its association with more high-resolution metrics of cerebrovascular reactivity.

### Supplementary Information

Additional file 1.

## Data Availability

Data is collected from Swedish medical institutions and contains private patient information, for access please contact Eric P Thelin for more details.
