# Supplementary material for: Critical thresholds of long-pressure reactivity index and impact of intracranial pressure monitoring methods in traumatic brain injury
Source: Crit Care. 2024 Jul 29;28:256. doi: 10.1186/s13054-024-05042-7 (PMC11285281; doi:10.1186/s13054-024-05042-7)
Supplement: Supplementary file 1 — Additional file 1. [file 13054_2024_5042_MOESM1_ESM.docx]

Contents

[Appendix A. Clinisoft Sampling Rate 3](#_Toc168526760)

[Appendix B. Imputation of the Data 4](#_Toc168526761)

[Appendix C. Yearly Patients 5](#_Toc168526762)

[Appendix D. First Days of Care and LPRx Results: Alive vs Dead 6](#_Toc168526763)

[Appendix E. First Days of Care and LPRx Results: Favorable vs Unfavorable 7](#_Toc168526764)

[Appendix F. LPRx for Each Day of Care: Alive vs Dead 8](#_Toc168526765)

[Appendix G. LPRx for Each Day of Care: Favorable vs Unfavorable 9](#_Toc168526766)

[Appendix H. Daily Scores 10](#_Toc168526767)

[Appendix I. Mann-Whitney U/Chi-Square Analysis of Physiologic and Demographic Data for Alive vs Dead and Favorable vs Unfavorable IPD Monitoring 14](#_Toc168526768)

[Appendix J. Mann-Whitney U/Chi-Square Analysis of Physiologic and Demographic Data for Alive vs Dead and Favorable vs Unfavorable EVD Monitoring 16](#_Toc168526769)

[Appendix K. ICP for Different ICP Monitoring Methods 18](#_Toc168526770)

[Appendix L. First Days of Care and ICP Results: Alive vs Dead 19](#_Toc168526771)

[Appendix M. First Days of Care and ICP Results: Favorable vs Unfavorable 20](#_Toc168526772)

[Appendix N. ICP for Each Day of Care: Alive vs Dead 21](#_Toc168526773)

[Appendix O. ICP for Each Day of Care: Favorable vs Unfavorable 22](#_Toc168526774)

[Appendix P. Non-Decompressive Patients 23](#_Toc168526775)

[Appendix Q. % Time LPRx over Thresholds Results: Alive vs Dead 24](#_Toc168526776)

[Appendix R. First Days of Care and % Time LPRx > 0.3 Results: Alive vs Dead 25](#_Toc168526777)

[Appendix S. First Days of Care and % Time LPRx > 0.3 Results: Favorable vs Unfavorable 27](#_Toc168526778)

[Appendix T. % Time LPRx > 0.3 for Each Day of Care: Alive vs Dead 28](#_Toc168526779)

[Appendix U. % Time LPRx > 0.3 for Each Day of Care: Favorable vs Unfavorable 29](#_Toc168526780)

[Appendix V. First Days of Care and % Time LPRx > 0 Results: Alive vs Dead 30](#_Toc168526781)

[Appendix W. First Days of Care and % Time LPRx > 0 Results: Favorable vs Unfavorable 31](#_Toc168526782)

[Appendix X. % Time LPRx > 0 for Each Day of Care: Alive vs Dead 32](#_Toc168526783)

[Appendix Y. % Time LPRx > 0 for Each Day of Care: Favorable vs Unfavorable 33](#_Toc168526784)

# Appendix A. Clinisoft Sampling Rate


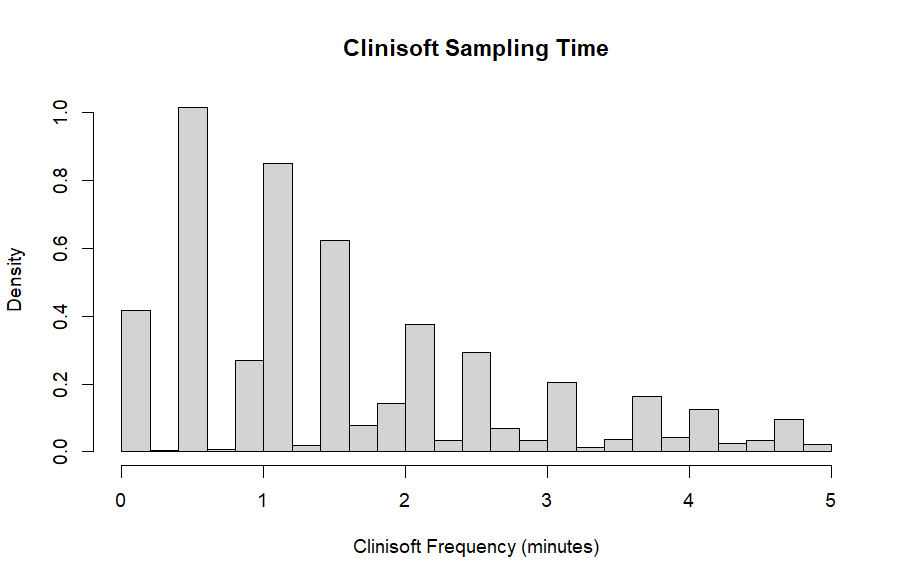


# Appendix B. Imputation of the Data

To transform the data to evenly sampled time series (at 1 minute), we imputed the data using locally weighted estimated scatterplot smoothing (LOWESS) over 20-minute windows, thus generating an imputed minute-by-minute median time series value for ICP and MAP. However, as the overall results were nearly identical (statistically similar for all key thresholds), the non-imputed data was demonstrated and referenced in the manuscript.

# Appendix C. Yearly Patients

| **Year** | **2006** | **2007** | **2008** | **2009** | **2010** | **2011** | **2012** | **2013** | **2014** | **2015** | **2016** | **2017** | **2018** | **2019** |
| --- | --- | --- | --- | --- | --- | --- | --- | --- | --- | --- | --- | --- | --- | --- |
| **Number of Patients** | 25 | 44 | 42 | 35 | 30 | 34 | 33 | 24 | 27 | 28 | 28 | 29 | 25 | 31 |
| **Alive Patients** | 17 (68%) | 32 (72%) | 37 (88.1%) | 27 (77.1%) | 26 (86.7%) | 29 (85.3%) | 27 (81.8%) | 19 (79.2%) | 23 (85.2%) | 23 (82.1%) | 24 (85.7%) | 23 (79%) | 20 (80%) | 24 (77%) |
| **Favorable Outcome** | 9 (36%) | 18 (41%) | 17 (40.5%) | 17 (48.6%) | 17 (56.7%) | 17 (50%) | 17 (51.5%) | 12 (50%) | 15 (55.6%) | 12 (42.9%) | 15 (53.6%) | 13 (44.8%) | 13 (52%) | 14 (45.2%) |
| **EVD Monitoring** | 18 (72%) | 26 (56%) | 25 (59.5%) | 19 (54.3%) | 21 (70%) | 23 (67.6%) | 15 (45.5%) | 17 (70.8%) | 16 (59.3%) | 18 (64.3%) | 8 (28.6%) | 8 (27.7%) | 8 (32%) | 6 (19.4%) |

The table subcategorise the data based on year of admission. EVD, external ventricular drain.

# Appendix D. First Days of Care and LPRx Results: Alive vs Dead


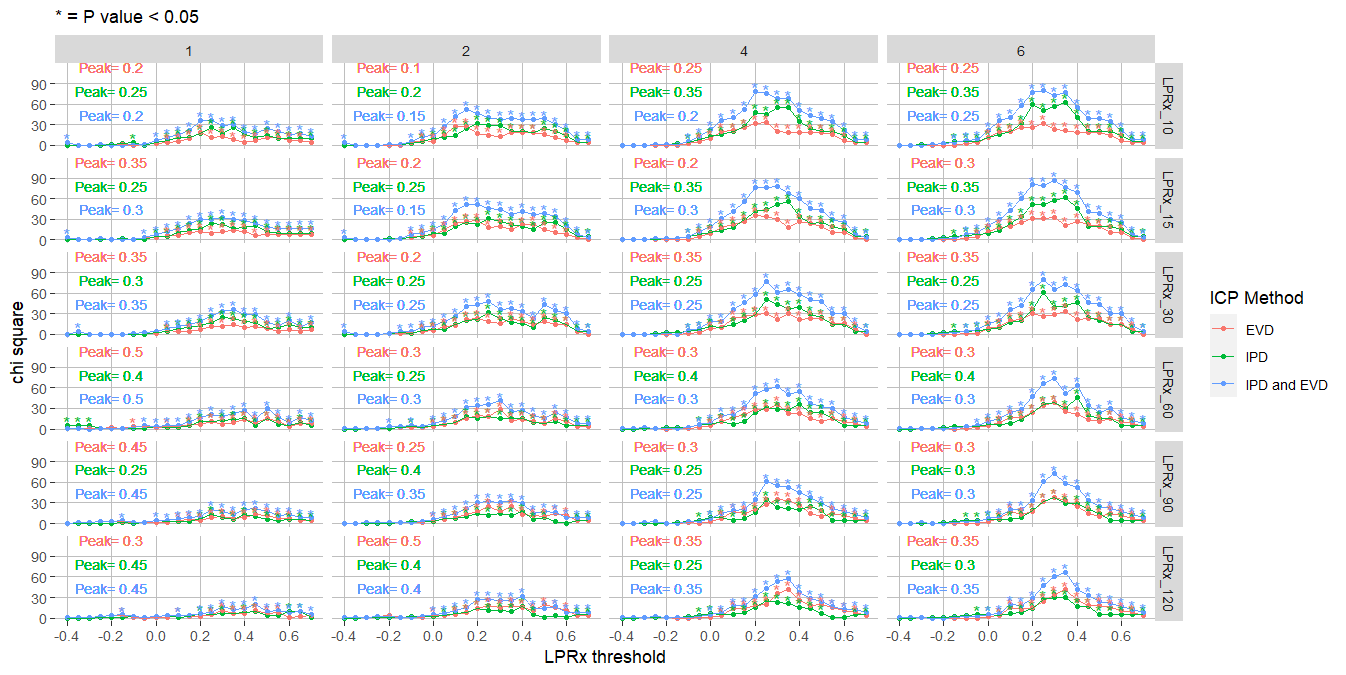


The figure displays a trend of the cumulative first days (1/2/4/6 days total) of care and the change in the resulting LPRx threshold. EVD, external ventricular drain; ICP, intracranial pressure; IPD, intraparenchymal monitoring; LPRx, long pressure reactivity;

# Appendix E. First Days of Care and LPRx Results: Favorable vs Unfavorable

­­­­
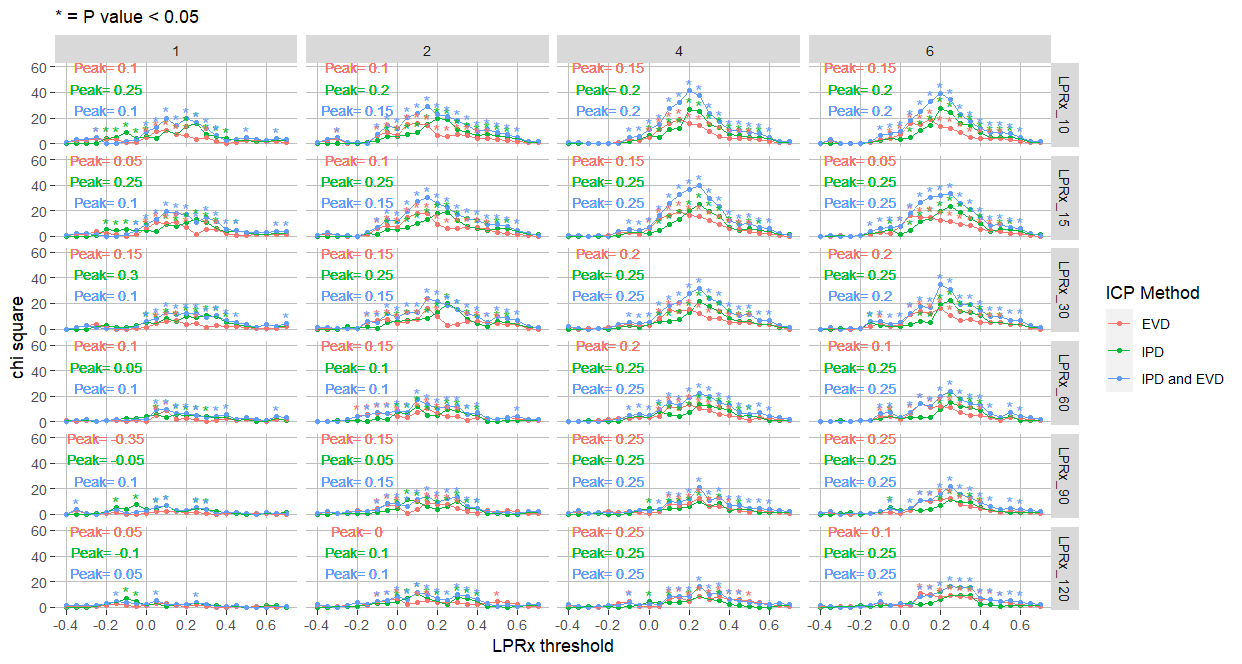
­­

The figure displays a trend of the cumulative first days (1/2/4/6 days total) of care and the change in the resulting LPRx threshold. EVD, external ventricular drain; ICP, intracranial pressure; IPD, intraparenchymal monitoring; LPRx, long pressure reactivity;

# Appendix F. LPRx for Each Day of Care: Alive vs Dead


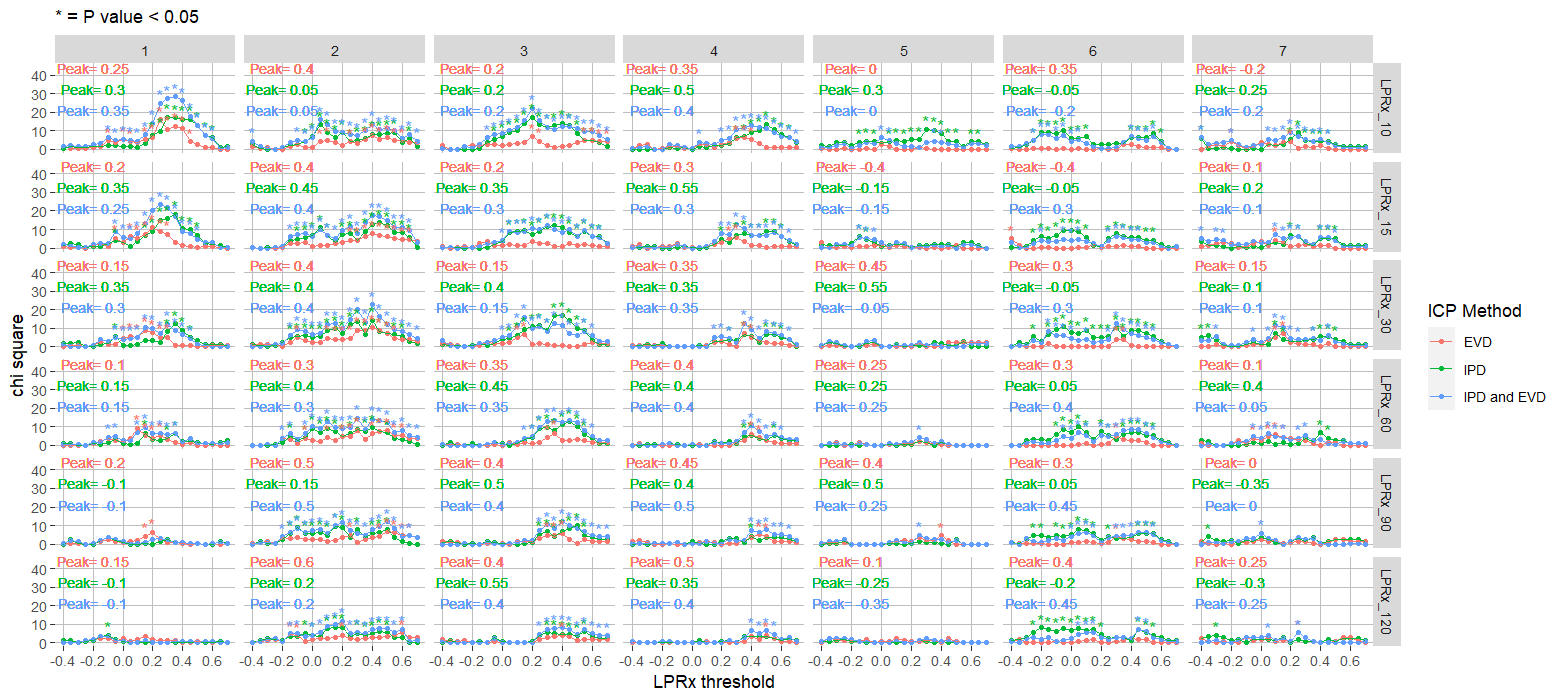


The figure displays a trend of a single day of care (day 1/2/3/4/5/6/7) and resulting LPRx threshold. Each day is a single 24 hour period from the initial start of recording (ie. day 1 = 0-24 hours of recording, day 2 = 25-48 hours of recording, etc.). EVD, external ventricular drain; ICP, intracranial pressure; IPD, intraparenchymal monitoring; LPRx, long pressure reactivity;

# Appendix G. LPRx for Each Day of Care: Favorable vs Unfavorable


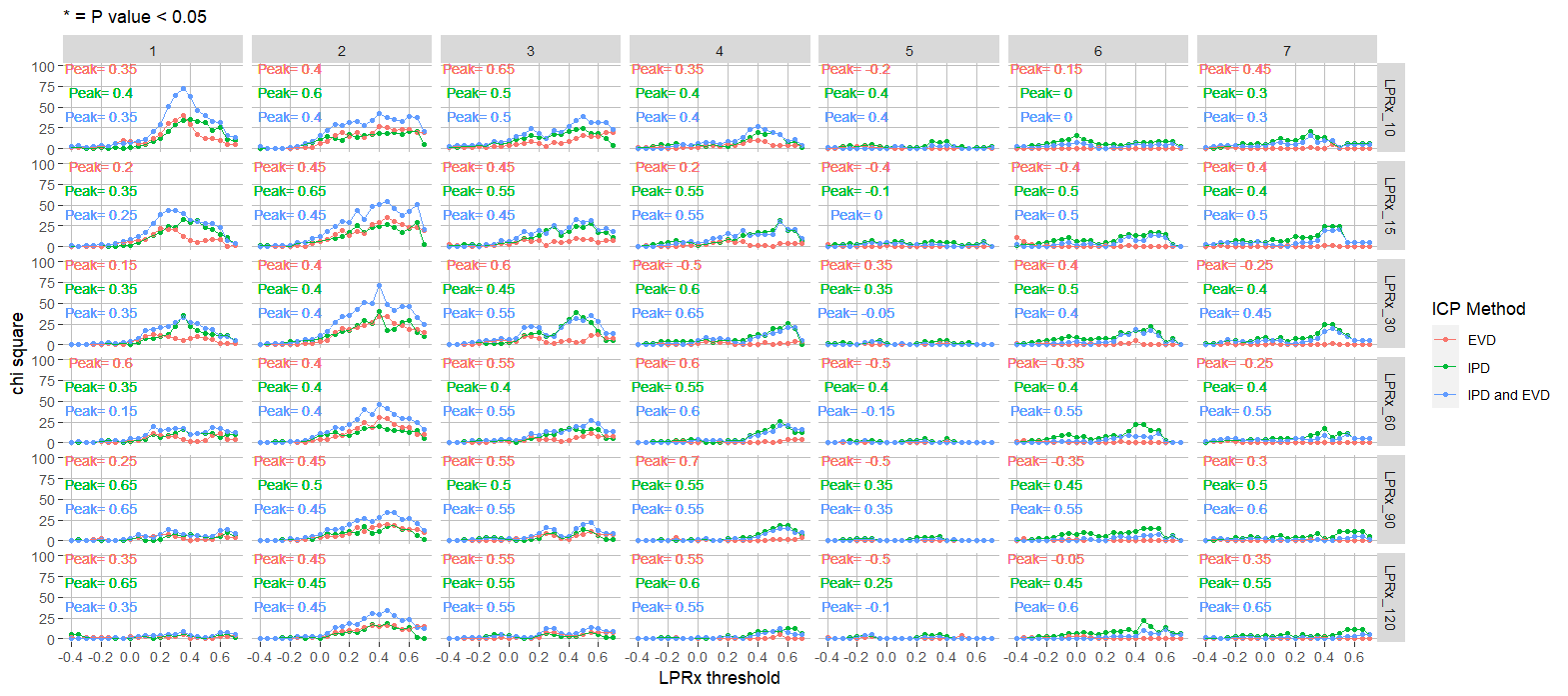


The figure displays a trend of a single day of care (day 1/2/3/4/5/6/7) and resulting LPRx threshold. Each day is a single 24 hour period from the initial start of recording (ie. day 1 = 0-24 hours of recording, day 2 = 25-48 hours of recording, etc.). EVD, external ventricular drain; ICP, intracranial pressure; IPD, intraparenchymal monitoring; LPRx, long pressure reactivity;

# Appendix H. Daily Scores

| **Day** | **1** | | | **2** | | | **3** | | |
| --- | --- | --- | --- | --- | --- | --- | --- | --- | --- |
| **Variable** | **Alive/Dead Outcome Groups** | | | **Alive/Dead Outcome Groups** | | | **Alive/Dead Outcome Groups** | | |
|  | **Dead Median (IQR)/ Number (%)** | **Alive Median (IQR)/ Number (%)** | **p value** | **Dead Median (IQR)/ Number (%)** | **Alive Median (IQR)/ Number (%)** | **p value** | **Dead Median (IQR)/ Number (%)** | **Alive Median (IQR)/ Number (%)** | **p value** |
| Number of Patients | 83 | 349 |  | 73 | 305 |  | 55 | 248 |  |
| Age (years) | 60 (44-69) | 50 (30-61) | **p<0.0001** | 60 (48-69) | 49 (30-61) | **p<0.0001** | 62 (52-70) | 49 (30-60) | **p<0.0001** |
| GCS Eye | 1 (1-1) | 1 (1-2) | **0.023** | 1 (1-1) | 1 (1-2) | 0.0474 | 1 (1-1) | 1 (1-2) | 0.1682 |
| GCS Motor | 2 (1-4) | 4 (2-5) | **p<0.0001** | 2 (1-4) | 4 (2-5) | **p<0.0001** | 3 (1-4) | 4 (2.25-5) | **0.0032** |
| GCS Verbal | 1 (1-1) | 1 (1-2) | **0.0033** | 1 (1-1) | 1 (1-2) | **0.0072** | 1 (1-1) | 1 (1-2) | 0.0563 |
| GCS | 5 (3-7.75) | 7 (5-10) | **p<0.001** | 5 (3-8) | 7 (5-10) | **p<0.001** | 5 (4-8) | 7 (5-9) | **0.0112** |
| Pupils Bilat Unreactive | 33 (39.8%) | 52 (14.9%) | **p<0.0001** | 27 (37%) | 46 (15.1%) | **p<0.0001** | 20 (36.4%) | 39 (15.7%) | **p<0.001** |
| Pupils Unilateral Unreactive | 11 (13.3%) | 49 (14%) | 0.853 | 9 (12.3%) | 45 (14.8%) | 0.5959 | 6 (10.9%) | 36 (14.5%) | 0.4853 |
| Pupils Bilat Reactive | 49 (59%) | 276 (79.1%) | **p<0.001** | 45 (61.6%) | 238 (78%) | **0.0038** | 33 (60%) | 189 (76.2%) | **0.0142** |
| Sex (Male) | 67 (80.7%) | 268 (76.8%) | 0.4413 | 62 (84.9%) | 235 (77%) | 0.1411 | 46 (83.6%) | 191 (77%) | 0.2832 |
| Hypoxia | 36 (43.4%) | 98 (28.1%) | **0.0069** | 31 (42.5%) | 88 (28.9%) | **0.0247** | 19 (34.5%) | 78 (31.5%) | 0.6577 |
| Hypotension | 31 (37.3%) | 110 (31.5%) | 0.3094 | 27 (37%) | 100 (32.8%) | 0.496 | 20 (36.4%) | 84 (33.9%) | 0.7259 |
| Marshall CT Score |  |  |  |  |  |  |  |  |  |
| V-VI | 50 (60.2%) | 183 (52.4%) | 0.2005 | 44 (60.3%) | 160 (52.5%) | 0.2297 | 35 (63.6%) | 124 (50%) | 0.0675 |
| IV | 3 (3.61%) | 13 (3.72%) | 0.963 | 3 (4.11%) | 12 (3.93%) | 0.9466 | 3 (5.45%) | 11 (4.44%) | 0.7468 |
| III | 14 (16.9%) | 59 (16.9%) | 0.994 | 12 (16.4%) | 50 (16.4%) | 0.9933 | 6 (10.9%) | 44 (17.7%) | 0.2181 |
| II | 16 (19.3%) | 100 (28.7%) | 0.0837 | 14 (19.2%) | 89 (29.2%) | 0.0852 | 11 (20%) | 72 (29%) | 0.1753 |
| I | 0 (0%) | 2 (0.573%) | 0.5429 | 0 (0%) | 2 (0.656%) | 0.5478 | 0 (0%) | 1 (0.403%) | 0.251 |
| Traumatic Subarachnoid or Intraventricular Hemorrhage | 70 (84.3%) | 285 (81.7%) | 0.568 | 61 (83.6%) | 249 (81.6%) | 0.7019 | 47 (85.5%) | 208 (83.9%) | 0.7725 |
| Epidural Hematoma | 3 (3.61%) | 63 (18.1%) | **0.001** | 0 (0%) | 56 (18.4%) | **p<0.001** | 0 (0%) | 47 (19%) | **0.0015** |
| Surgical Evacuation of Lesions | 49 (59%) | 209 (59.9%) | 0.8878 | 43 (58.9%) | 179 (58.7%) | 0.9738 | 35 (63.6%) | 146 (58.9%) | 0.5158 |
| Decompressive Craniectomy Primary | 13 (15.7%) | 22 (6.3%) | **0.005** | 11 (15.1%) | 16 (5.25%) | **0.0035** | 6 (10.9%) | 15 (6.05%) | 0.2005 |
| Decompressive Craniectomy Secondary | 3 (3.61%) | 6 (1.72%) | 0.2786 | 3 (4.11%) | 4 (1.31%) | 0.1122 | 3 (5.45%) | 4 (1.61%) | 0.0873 |
| ICU Length of Stay (Days) | 5.79 (2.82-13.2) | 11 (5.5-16) | **p<0.001** | 6 (3-13.5) | 11 (5.83-16) | **p<0.001** | 10 (4.1-15.2) | 12 (7.96-17) | **0.0093** |
| MAP (mmHg) | 76.6 (71.9-82.3) | 75.3 (71.1-79.9) | 0.1172 | 76.8 (72.5-81.3) | 77 (72.6-81.8) | 0.6851 | 75 (70.8-79.5) | 77.1 (73.3-82.7) | **0.0209** |
| ICP (mmHg) | 15.4 (11.1-22) | 11 (7.84-14.3) | **p<0.0001** | 16.4 (12.4-24.8) | 12.2 (8.62-14.8) | **p<0.0001** | 14.3 (10.4-18) | 11.6 (8.94-14.7) | **0.0011** |
| CPP (mmHg) | 59.5 (53.7-66.3) | 64.3 (59.6-68.9) | **p<0.0001** | 60.6 (53.4-65) | 64.9 (60.7-70.4) | **p<0.0001** | 60.8 (55.7-64.8) | 65.9 (60.8-70.7) | **p<0.0001** |
| LPRx_10 (au) | 0.219 (0.0587-0.338) | 0.0676 (-0.0371-0.171) | **p<0.0001** | 0.212 (0.0796-0.426) | 0.0248 (-0.101-0.14) | **p<0.0001** | 0.147 (0.00775-0.407) | -0.00791 (-0.139-0.114) | **p<0.0001** |
| LPRx_15 (au) | 0.222 (0.0519-0.338) | 0.0657 (-0.0298-0.165) | **p<0.0001** | 0.216 (0.0894-0.425) | 0.0244 (-0.107-0.152) | **p<0.0001** | 0.155 (0.0237-0.362) | -0.0101 (-0.151-0.126) | **p<0.0001** |
| LPRx_20 (au) | 0.21 (0.0852-0.32) | 0.0683 (-0.0265-0.173) | **p<0.0001** | 0.221 (0.07-0.408) | 0.0231 (-0.114-0.147) | **p<0.0001** | 0.154 (0.00951-0.401) | -0.00828 (-0.149-0.134) | **p<0.0001** |
| LPRx_30 (au) | 0.212 (0.0841-0.341) | 0.0784 (-0.0334-0.192) | **p<0.0001** | 0.227 (0.108-0.442) | 0.0366 (-0.104-0.169) | **p<0.0001** | 0.165 (0.00885-0.43) | 0.00253 (-0.136-0.137) | **p<0.0001** |
| LPRx_60 (au) | 0.196 (0.0852-0.326) | 0.0889 (-0.0181-0.221) | **p<0.0001** | 0.219 (0.0843-0.427) | 0.0697 (-0.0852-0.196) | **p<0.0001** | 0.155 (0.0052-0.344) | 0.0465 (-0.0999-0.176) | **p<0.001** |
| LPRx_90 (au) | 0.192 (0.0718-0.345) | 0.108 (-0.00412-0.231) | **p<0.001** | 0.236 (0.0667-0.425) | 0.0909 (-0.0672-0.227) | **p<0.0001** | 0.139 (-0.0107-0.351) | 0.061 (-0.093-0.202) | **0.0041** |
| LPRx_120 (au) | 0.191 (0.0824-0.347) | 0.12 (0.00277-0.251) | **0.0012** | 0.237 (0.0822-0.433) | 0.112 (-0.0507-0.236) | **p<0.0001** | 0.168 (0.00454-0.343) | 0.0757 (-0.0656-0.225) | **0.0096** |

| **Day** | **4** | | | **5** | | | **6** | | |
| --- | --- | --- | --- | --- | --- | --- | --- | --- | --- |
| **Variable** | **Alive/Dead Outcome Groups** | | | **Alive/Dead Outcome Groups** | | | **Alive/Dead Outcome Groups** | | |
|  | **Dead Median (IQR)/ Number (%)** | **Alive Median (IQR)/ Number (%)** | **p value** | **Dead Median (IQR)/ Number (%)** | **Alive Median (IQR)/ Number (%)** | **p value** | **Dead Median (IQR)/ Number (%)** | **Alive Median (IQR)/ Number (%)** | **p value** |
| Number of Patients | 43 | 223 |  | 36 | 191 |  | 29 | 166 |  |
| Age (years) | 63 (52.5-69.5) | 47 (29.5-59) | **p<0.0001** | 63 (56-70.2) | 47 (29-59.5) | **p<0.0001** | 63 (59-69) | 46 (29-58.8) | **p<0.0001** |
| GCS Eye | 1 (1-1.5) | 1 (1-2) | 0.2957 | 1 (1-1) | 1 (1-2) | **0.0834** | 1 (1-1) | 1 (1-2) | 0.1123 |
| GCS Motor | 2 (1-4) | 4 (2-5) | **0.012** | 3 (1.5-4) | 4 (3-5) | **0.0067** | 2.5 (1.25-4) | 4 (3-5) | **0.0107** |
| GCS Verbal | 1 (1-1) | 1 (1-2) | 0.101 | 1 (1-1) | 1 (1-2) | **0.0543** | 1 (1-1.75) | 1 (1-2) | 0.1146 |
| GCS | 5 (3.5-7.5) | 7 (5-9) | **0.0189** | 6 (4-8) | 7 (5-10) | **0.0168** | 6 (4-8) | 7 (5-10) | **0.0141** |
| Pupils Bilat Unreactive | 13 (30.2%) | 40 (17.9%) | 0.0653 | 9 (25%) | 29 (15.2%) | 0.1493 | 8 (27.6%) | 26 (15.7%) | 0.12 |
| Pupils Unilateral Unreactive | 4 (9.3%) | 36 (16.1%) | 0.2521 | 4 (11.1%) | 29 (15.2%) | 0.5273 | 3 (10.3%) | 25 (15.1%) | 0.507 |
| Pupils Bilat Reactive | 30 (69.8%) | 165 (74%) | 0.5681 | 27 (75%) | 147 (77%) | 0.8003 | 22 (75.9%) | 129 (77.7%) | 0.8284 |
| Sex (Male) | 36 (83.7%) | 168 (75.3%) | 0.2352 | 30 (83.3%) | 147 (77%) | 0.3997 | 23 (79.3%) | 128 (77.1%) | 0.7959 |
| Hypoxia | 18 (41.9%) | 72 (32.3%) | 0.2258 | 17 (47.2%) | 58 (30.4%) | 0.0493 | 13 (44.8%) | 50 (30.1%) | 0.1196 |
| Hypotension | 16 (37.2%) | 71 (31.8%) | 0.4935 | 15 (41.7%) | 63 (33%) | 0.3162 | 13 (44.8%) | 55 (33.1%) | 0.2247 |
| Marshall CT Score |  |  |  |  |  |  |  |  |  |
| V-VI | 28 (65.1%) | 109 (48.9%) | 0.0517 | 24 (66.7%) | 95 (49.7%) | 0.0629 | 20 (69%) | 83 (50%) | 0.06 |
| IV | 3 (6.98%) | 10 (4.48%) | 0.4903 | 2 (5.56%) | 9 (4.71%) | 0.8321 | 1 (3.45%) | 8 (4.82%) | 0.7498 |
| III | 1 (2.33%) | 40 (17.9%) | **0.0096** | 1 (2.78%) | 38 (19.9%) | **0.0128** | 1 (3.45%) | 32 (19.3%) | **0.0367** |
| II | 11 (25.6%) | 67 (30%) | 0.5577 | 9 (25%) | 52 (27.2%) | 0.7842 | 7 (24.1%) | 46 (27.7%) | 0.6923 |
| I | 0 (0%) | 1 (0.448%) | 0.203 | 0 (0%) | 1 (0.524%) | 0.1971 | 0 (0%) | 1 (0.602%) | 0.175 |
| Traumatic Subarachnoid or Intraventricular Hemorrhage | 36 (83.7%) | 193 (86.5%) | 0.6258 | 29 (80.6%) | 165 (86.4%) | 0.3648 | 24 (82.8%) | 143 (86.1%) | 0.6344 |
| Epidural Hematoma | 0 (0%) | 39 (17.5%) | **0.0099** | 0 (0%) | 35 (18.3%) | **0.0174** | 0 (0%) | 31 (18.7%) | 0.0371 |
| Surgical Evacuation of Lesions | 28 (65.1%) | 127 (57%) | 0.3216 | 22 (61.1%) | 112 (58.6%) | 0.7837 | 20 (69%) | 99 (59.6%) | 0.3443 |
| Decompressive Craniectomy Primary | 5 (11.6%) | 16 (7.17%) | 0.3235 | 4 (11.1%) | 14 (7.33%) | 0.4439 | 3 (10.3%) | 13 (7.83%) | 0.6526 |
| Decompressive Craniectomy Secondary | 3 (6.98%) | 4 (1.79%) | 0.0528 | 3 (8.33%) | 4 (2.09%) | **0.048** | 3 (10.3%) | 4 (2.41%) | **0.035** |
| ICU Length of Stay (Days) | 12 (5.9-16.2) | 13.2 (8.64-17.5) | 0.0813 | 13 (9.85-17) | 14.6 (9.41-18.2) | 0.3803 | 13 (10-17) | 15 (10.3-19) | 0.1277 |
| MAP (mmHg) | 77.4 (71.6-83.4) | 78.4 (74.4-83.7) | 0.069 | 79.5 (74.1-82.1) | 78.4 (74.2-84.1) | 0.841 | 77.5 (74.2-85.7) | 79 (74.9-83.4) | 0.8739 |
| ICP (mmHg) | 14.2 (11.4-17.4) | 11.7 (8.87-14.7) | **0.0023** | 12.4 (9.53-17.2) | 11.9 (8.78-15.2) | 0.2619 | 13.4 (10.3-17.7) | 12.5 (9.22-15.4) | 0.1081 |
| CPP (mmHg) | 62.6 (57.5-66.1) | 66.6 (62.1-72.5) | **p<0.0001** | 63.7 (60.8-68.9) | 66.2 (62.5-71) | 0.0746 | 63.8 (57.7-68.5) | 66.5 (62.6-71) | **0.0314** |
| LPRx_10 (au) | 0.067 (-0.0647-0.279) | -0.0218 (-0.149-0.0918) | **0.0037** | -0.0353 (-0.126-0.181) | -0.0557 (-0.202-0.094) | 0.2007 | 0.0363 (-0.0561-0.186) | -0.0433 (-0.246-0.0659) | **0.0199** |
| LPRx_15 (au) | 0.0884 (-0.0911-0.261) | -0.0273 (-0.166-0.0943) | **0.0035** | -0.0259 (-0.139-0.145) | -0.0704 (-0.218-0.0925) | 0.2212 | 0.0471 (-0.0726-0.153) | -0.0522 (-0.229-0.0672) | **0.0284** |
| LPRx_20 (au) | 0.0892 (-0.12-0.249) | -0.032 (-0.171-0.0919) | **0.011** | -0.0325 (-0.159-0.167) | -0.0609 (-0.214-0.0962) | 0.3278 | 0.0243 (-0.107-0.184) | -0.0726 (-0.229-0.0846) | 0.055 |
| LPRx_30 (au) | 0.0833 (-0.124-0.213) | -0.0157 (-0.158-0.117) | **0.035** | -0.00974 (-0.132-0.156) | -0.0548 (-0.219-0.0927) | 0.306 | 0.00977 (-0.168-0.224) | -0.0611 (-0.228-0.0892) | 0.073 |
| LPRx_60 (au) | 0.0793 (-0.127-0.225) | 0.0102 (-0.137-0.148) | **0.0964** | -0.0401 (-0.13-0.201) | -0.0182 (-0.172-0.127) | 0.5407 | 0.00849 (-0.0916-0.28) | -0.0358 (-0.185-0.135) | 0.1718 |
| LPRx_90 (au) | 0.114 (-0.0597-0.246) | 0.035 (-0.11-0.179) | 0.1125 | -0.0161 (-0.135-0.189) | -0.00337 (-0.141-0.165) | 0.5884 | 0.0654 (-0.148-0.287) | -0.0223 (-0.162-0.159) | 0.2305 |
| LPRx_120 (au) | 0.135 (-0.071-0.249) | 0.0571 (-0.0976-0.194) | 0.1358 | -0.00719 (-0.116-0.205) | 0.00989 (-0.134-0.189) | 0.6061 | 0.0391 (-0.113-0.264) | 0.00735 (-0.148-0.176) | 0.2324 |

*Au = arbitrary units, CPP = cerebral perfusion pressure, CT = computed tomography, GCS = Glasgow Coma Scale, ICP = intracranial pressure, ICU = intensive care unit, IQR = interquartile range, LPRx = long pressure reactivity index, MAP = mean arterial pressure, mmHg= millimeters of mercury.*

# Appendix I. Mann-Whitney U/Chi-Square Analysis of Physiologic and Demographic Data for Alive vs Dead and Favorable vs Unfavorable IPD Monitoring

| **Variable** | **Alive/Dead Outcome Groups** | | | **Favorable/Unfavorable Outcome Groups** | | |
| --- | --- | --- | --- | --- | --- | --- |
|  | **Dead Median (IQR)/ Number (%)** | **Alive Median (IQR)/ Number (%)** | **p value** | **Unfavorable Median (IQR)/ Number (%)** | **Favorable Median (IQR)/ Number (%)** | **p value** |
| Number of Patients | 36 | 171 |  | 93 | 114 |  |
| Age (years) | 60.5 (50.8-70.2) | 48 (29-60.5) | **p<0.001** | 59 (46-69) | 42 (28-58) | **p<0.0001** |
| GCS Eye | 1 (1-1) | 1 (1-3) | 0.1126 | 1 (1-2) | 1 (1-3) | 0.0662 |
| GCS Motor | 2 (1-4) | 4 (2-5) | **0.0017** | 3 (1-5) | 5 (3-5) | **p<0.001** |
| GCS Verbal | 1 (1-1) | 1 (1-2.25) | **0.0106** | 1 (1-2) | 1 (1-3) | **0.0039** |
| GCS | 5 (3-8.5) | 7 (5-10) | **0.0067** | 6 (3-8) | 8 (5-11) | **p<0.001** |
| Pupils Bilat Unreactive | 13 (36.1%) | 25 (14.6%) | **0.0025** | 25 (26.9%) | 13 (11.4%) | **0.0043** |
| Pupils Unilateral Unreactive | 4 (11.1%) | 23 (13.5%) | 0.7075 | 12 (12.9%) | 15 (13.2%) | 0.9585 |
| Pupils Bilat Reactive | 23 (63.9%) | 133 (77.8%) | 0.0799 | 60 (64.5%) | 96 (84.2%) | **0.0011** |
| Sex (Male) | 29 (80.6%) | 126 (73.7%) | 0.3899 | 69 (74.2%) | 86 (75.4%) | 0.8388 |
| Hypoxia | 15 (41.7%) | 42 (24.6%) | **0.0374** | 32 (34.4%) | 25 (21.9%) | **0.0463** |
| Hypotension | 13 (36.1%) | 50 (29.2%) | 0.4177 | 32 (34.4%) | 31 (27.2%) | 0.2635 |
| Marshall CT Score |  |  |  |  |  |  |
| V-VI | 24 (66.7%) | 98 (57.3%) | 0.3016 | 58 (62.4%) | 64 (56.1%) | 0.367 |
| IV | 3 (8.33%) | 3 (1.75%) | **0.0333** | 4 (4.3%) | 2 (1.75%) | 0.2803 |
| III | 6 (16.7%) | 23 (13.5%) | 0.616 | 12 (12.9%) | 17 (14.9%) | 0.6808 |
| II | 3 (8.33%) | 50 (29.2%) | **0.0092** | 19 (20.4%) | 34 (29.8%) | 0.1247 |
| I | 0 (0%) | 1 (0.585%) | 0.2359 | 0 (0%) | 1 (0.877%) | 0.8965 |
| Traumatic Subarachnoid or Intraventricular Hemorrhage | 28 (77.8%) | 135 (78.9%) | 0.8781 | 78 (83.9%) | 85 (74.6%) | 0.1046 |
| Epidural Hematoma | 1 (2.78%) | 38 (22.2%) | **0.0069** | 6 (6.45%) | 33 (28.9%) | **p<0.0001** |
| Surgical Evacuation of Lesions | 23 (63.9%) | 106 (62%) | 0.8325 | 57 (61.3%) | 72 (63.2%) | 0.7843 |
| Decompressive Craniectomy Primary | 4 (11.1%) | 8 (4.68%) | 0.1352 | 9 (9.68%) | 3 (2.63%) | **0.0316** |
| Decompressive Craniectomy Secondary | 0 (0%) | 1 (0.585%) | 0.2359 | 0 (0%) | 1 (0.877%) | 0.8965 |
| ICU Length of Stay (Days) | 4.64 (2.47-12.4) | 10.1 (4.94-15.5) | 0.0132 | 8.87 (3.64-15) | 10 (4.59-15.5) | 0.2922 |
| MAP (mmHg) | 76.2 (73.8-83) | 78.5 (73.2-82.7) | 0.8796 | 76.7 (73.5-83) | 78.6 (73.4-82.1) | 0.8657 |
| ICP (mmHg) | 17.5 (12.7-35.1) | 11.4 (8.66-14.3) | **p<0.0001** | 11.8 (8.68-16.7) | 12.1 (9.02-14.6) | 0.4311 |
| CPP (mmHg) | 59.4 (45.9-64.2) | 66 (62.2-71.2) | **p<0.0001** | 64.7 (59.3-70.7) | 65.5 (61.8-70.7) | 0.1848 |
| LPRx_10 (au) | 0.265 (0.07-0.447) | 0.0311 (-0.0834-0.126) | **p<0.0001** | 0.0811 (-0.0292-0.261) | 0.0335 (-0.0857-0.113) | **0.0014** |
| LPRx_15 (au) | 0.268 (0.0657-0.457) | 0.0397 (-0.0951-0.125) | **p<0.0001** | 0.0884 (-0.0197-0.269) | 0.0427 (-0.0937-0.116) | **0.0017** |
| LPRx_20 (au) | 0.279 (0.0594-0.443) | 0.0411 (-0.0863-0.134) | **p<0.0001** | 0.0858 (-0.0153-0.278) | 0.042 (-0.0906-0.123) | **0.0026** |
| LPRx_30 (au) | 0.305 (0.0815-0.456) | 0.0459 (-0.0813-0.15) | **p<0.0001** | 0.115 (-0.0235-0.295) | 0.0606 (-0.0845-0.144) | **0.0016** |
| LPRx_60 (au) | 0.321 (0.105-0.449) | 0.0789 (-0.0424-0.188) | **p<0.0001** | 0.128 (-9.42e-05-0.323) | 0.0765 (-0.0447-0.183) | **0.0039** |
| LPRx_90 (au) | 0.319 (0.131-0.442) | 0.101 (-0.0229-0.211) | **p<0.0001** | 0.157 (0.038-0.341) | 0.0969 (-0.0234-0.207) | **0.0038** |
| LPRx_120 (au) | 0.303 (0.106-0.417) | 0.117 (0.000489-0.225) | **p<0.0001** | 0.179 (0.0499-0.336) | 0.113 (-0.0141-0.22) | **0.0057** |

*Au = arbitrary units, CPP = cerebral perfusion pressure, CT = computed tomography, GCS = Glasgow Coma Scale, ICP = intracranial pressure, ICU = intensive care unit, IPD, intraparenchymal monitoring; IQR = interquartile range, LPRx = long pressure reactivity index, MAP = mean arterial pressure, mmHg= millimeters of mercury.*

# Appendix J. Mann-Whitney U/Chi-Square Analysis of Physiologic and Demographic Data for Alive vs Dead and Favorable vs Unfavorable EVD Monitoring

| **Variable** | **Alive/Dead Outcome Groups** | | | **Favorable/Unfavorable Outcome Groups** | | |
| --- | --- | --- | --- | --- | --- | --- |
|  | **Dead Median (IQR)/ Number (%)** | **Alive Median (IQR)/ Number (%)** | **p value** | **Unfavorable Median (IQR)/ Number (%)** | **Favorable Median (IQR)/ Number (%)** | **p value** |
| Number of Patients | 48 | 180 |  | 128 | 100 |  |
| Age (years) | 58 (41.8-69) | 51 (34-61.2) | **0.0203** | 58 (41.8-66) | 45.5 (29-56.2) | **p<0.0001** |
| GCS Eye | 1 (1-1) | 1 (1-2) | 0.1014 | 1 (1-2) | 1 (1-3) | 0.0587 |
| GCS Motor | 2 (1-4) | 4 (2-5) | **0.0011** | 3 (2-4) | 4 (2.25-5) | **0.0023** |
| GCS Verbal | 1 (1-1) | 1 (1-2) | 0.1013 | 1 (1-2) | 1 (1-2) | 0.1137 |
| GCS | 5 (3.75-7) | 7 (5-10) | **0.0056** | 6 (4-8) | 7 (5-10) | **0.014** |
| Pupils Bilat Unreactive | 20 (41.7%) | 27 (15%) | **p<0.001** | 33 (25.8%) | 14 (14%) | **0.0296** |
| Pupils Unilateral Unreactive | 7 (14.6%) | 27 (15%) | 0.9443 | 21 (16.4%) | 13 (13%) | 0.4757 |
| Pupils Bilat Reactive | 27 (56.2%) | 144 (80%) | **p<0.001** | 86 (67.2%) | 85 (85%) | **0.0021** |
| Sex (Male) | 39 (81.2%) | 144 (80%) | 0.8484 | 102 (79.7%) | 81 (81%) | 0.8064 |
| Hypoxia | 21 (43.8%) | 57 (31.7%) | 0.1181 | 53 (41.4%) | 25 (25%) | **0.0098** |
| Hypotension | 18 (37.5%) | 60 (33.3%) | 0.5906 | 49 (38.3%) | 29 (29%) | 0.1439 |
| Marshall CT Score |  |  |  |  |  |  |
| V-VI | 26 (54.2%) | 85 (47.2%) | 0.3942 | 65 (50.8%) | 46 (46%) | 0.4752 |
| IV | 0 (0%) | 10 (5.56%) | 0.3103 | 4 (3.12%) | 6 (6%) | 0.2952 |
| III | 9 (18.8%) | 36 (20%) | 0.8484 | 24 (18.8%) | 21 (21%) | 0.6736 |
| II | 13 (27.1%) | 52 (28.9%) | 0.8072 | 38 (29.7%) | 27 (27%) | 0.6572 |
| I | 0 (0%) | 1 (0.556%) | 0.3266 | 1 (0.781%) | 0 (0%) | 0.8713 |
| Traumatic Subarachnoid or Intraventricular Hemorrhage | 43 (89.6%) | 152 (84.4%) | 0.3707 | 112 (87.5%) | 83 (83%) | 0.3398 |
| Epidural Hematoma | 3 (6.25%) | 26 (14.4%) | 0.1314 | 10 (7.81%) | 19 (19%) | **0.0121** |
| Surgical Evacuation of Lesions | 27 (56.2%) | 104 (57.8%) | 0.8506 | 72 (56.2%) | 59 (59%) | 0.6784 |
| Decompressive Craniectomy Primary | 9 (18.8%) | 14 (7.78%) | **0.0254** | 15 (11.7%) | 8 (8%) | 0.3569 |
| Decompressive Craniectomy Secondary | 3 (6.25%) | 5 (2.78%) | 0.248 | 8 (6.25%) | 0 (0%) | **0.0427** |
| ICU Length of Stay (Days) | 9.45 (3-13.6) | 12.2 (6.5-17.4) | **0.0022** | 12.9 (6-17) | 10.6 (5.36-15.3) | 0.2281 |
| MAP (mmHg) | 77.3 (75-80.5) | 78.3 (75.1-82.6) | 0.2913 | 77.7 (74.7-81.1) | 78.6 (75.3-82.8) | 0.1594 |
| ICP (mmHg) | 14.4 (11.9-25.2) | 11.1 (8.36-13.3) | **p<0.0001** | 12 (8.73-15.3) | 11 (8.42-13.2) | **0.0121** |
| CPP (mmHg) | 60.6 (51.2-65.9) | 67.5 (63.5-71.9) | **p<0.0001** | 65.1 (60.1-69.5) | 68.3 (64.5-72.3) | **p<0.001** |
| LPRx_10 (au) | 0.185 (0.0305-0.362) | 0.0276 (-0.0687-0.133) | **p<0.0001** | 0.0907 (-0.0432-0.22) | 0.00168 (-0.0724-0.116) | **p<0.001** |
| LPRx_15 (au) | 0.181 (0.0341-0.342) | 0.0169 (-0.0762-0.128) | **p<0.0001** | 0.0753 (-0.0465-0.219) | 0.0074 (-0.0759-0.106) | **0.0011** |
| LPRx_20 (au) | 0.177 (0.0469-0.325) | 0.014 (-0.0797-0.114) | **p<0.0001** | 0.0722 (-0.045-0.223) | 0.00423 (-0.0878-0.0887) | **p<0.001** |
| LPRx_30 (au) | 0.165 (0.0281-0.339) | 0.0149 (-0.0792-0.127) | **p<0.0001** | 0.075 (-0.0475-0.235) | 0.00816 (-0.0857-0.0929) | **0.0035** |
| LPRx_60 (au) | 0.146 (0.00824-0.326) | 0.0186 (-0.0784-0.127) | **p<0.0001** | 0.072 (-0.0398-0.221) | -0.00384 (-0.0962-0.109) | **0.0022** |
| LPRx_90 (au) | 0.151 (0.0133-0.348) | 0.0308 (-0.0611-0.139) | **p<0.001** | 0.068 (-0.0199-0.213) | 0.0134 (-0.0727-0.132) | **0.0142** |
| LPRx_120 (au) | 0.154 (0.0309-0.348) | 0.0321 (-0.05-0.146) | **p<0.001** | 0.0726 (-0.0117-0.191) | 0.0131 (-0.0617-0.135) | **0.0104** |

*Au = arbitrary units, CPP = cerebral perfusion pressure, CT = computed tomography, EVD = external ventricular drain, GCS = Glasgow Coma Scale, ICP = intracranial pressure, ICU = intensive care unit, IQR = interquartile range, LPRx = long pressure reactivity index, MAP = mean arterial pressure, mmHg= millimeters of mercury.*

# Appendix K. ICP for Different ICP Monitoring Methods


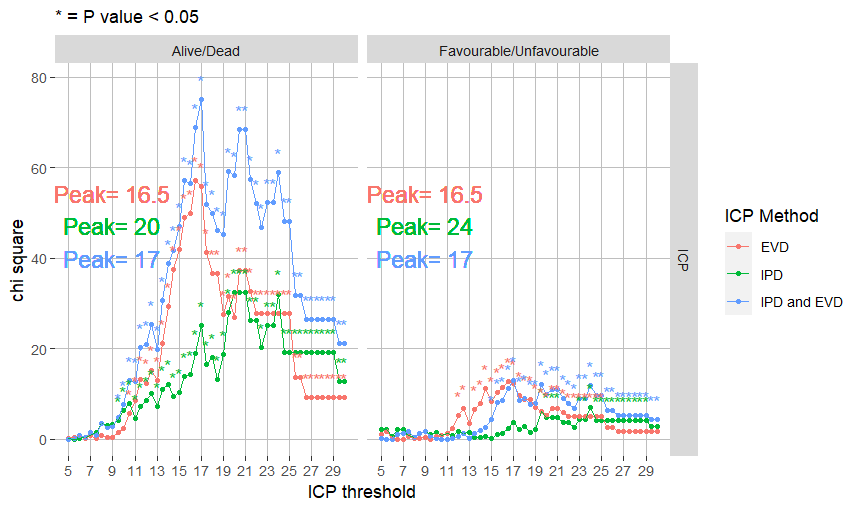


The figure displays a trend of ICP and the resulting thresholds with for all recording periods for different ICP monitoring methods. EVD, external ventricular drain; ICP, intracranial pressure; IPD, intraparenchymal monitoring;

# Appendix L. First Days of Care and ICP Results: Alive vs Dead


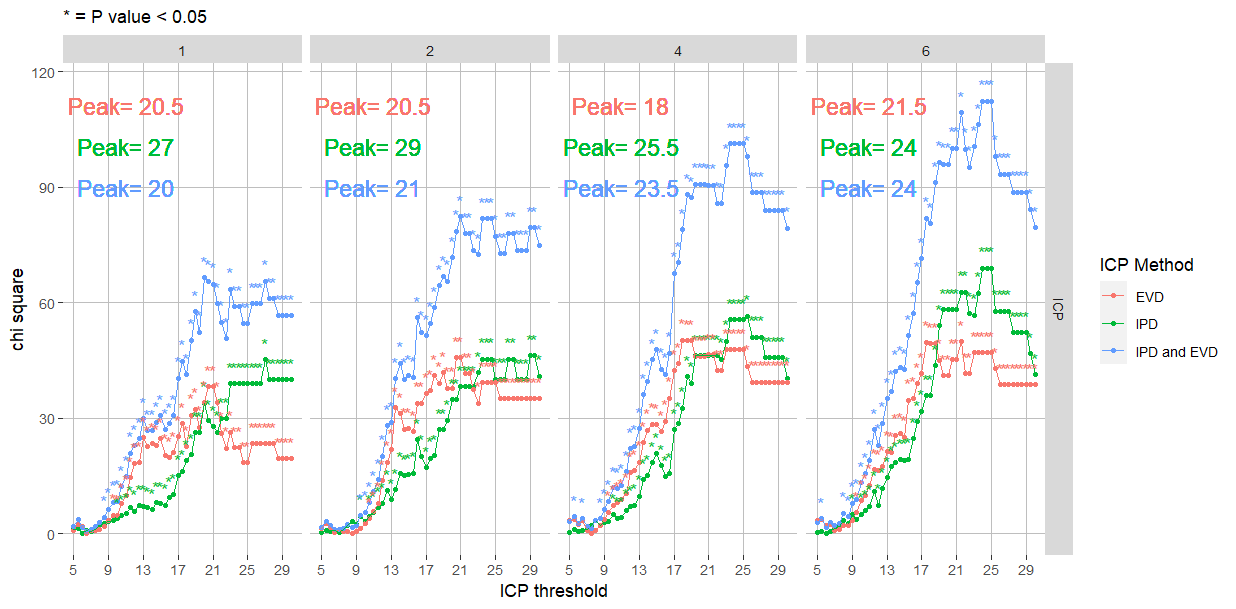


The figure displays a trend of the cumulative first days (1/2/4/6 days total) of care and the change in the resulting ICP threshold. EVD, external ventricular drain; ICP, intracranial pressure; IPD, intraparenchymal monitoring;

# Appendix M. First Days of Care and ICP Results: Favorable vs Unfavorable


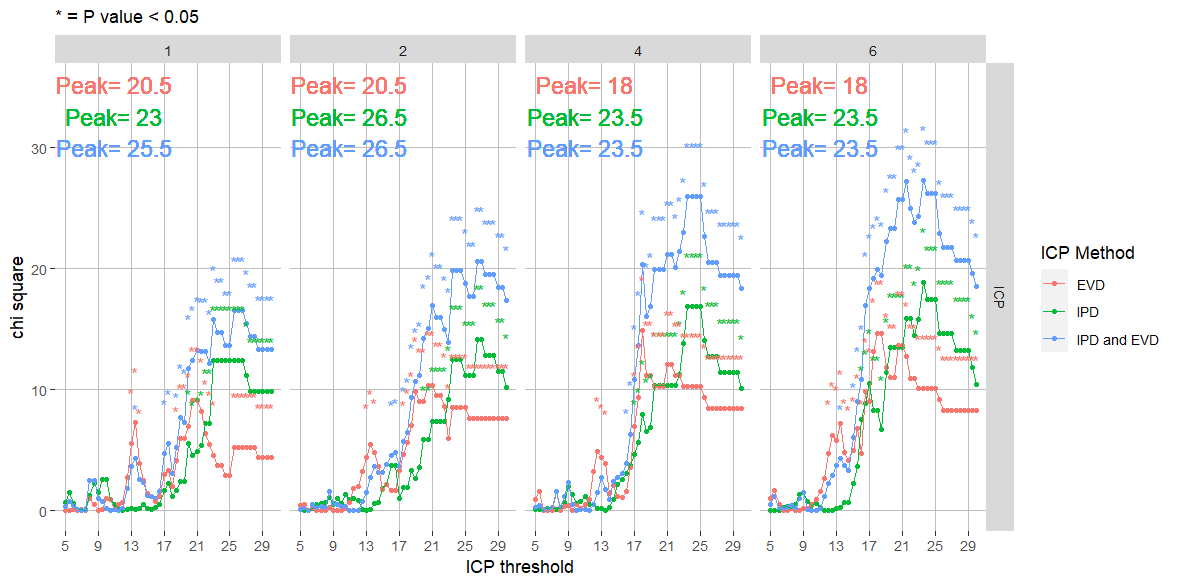


The figure displays a trend of the cumulative first days (1/2/4/6 days total) of care and the change in the resulting ICP threshold EVD, external ventricular drain; ICP, intracranial pressure; IPD, intraparenchymal monitoring; LPRx, long pressure reactivity;

# Appendix N. ICP for Each Day of Care: Alive vs Dead


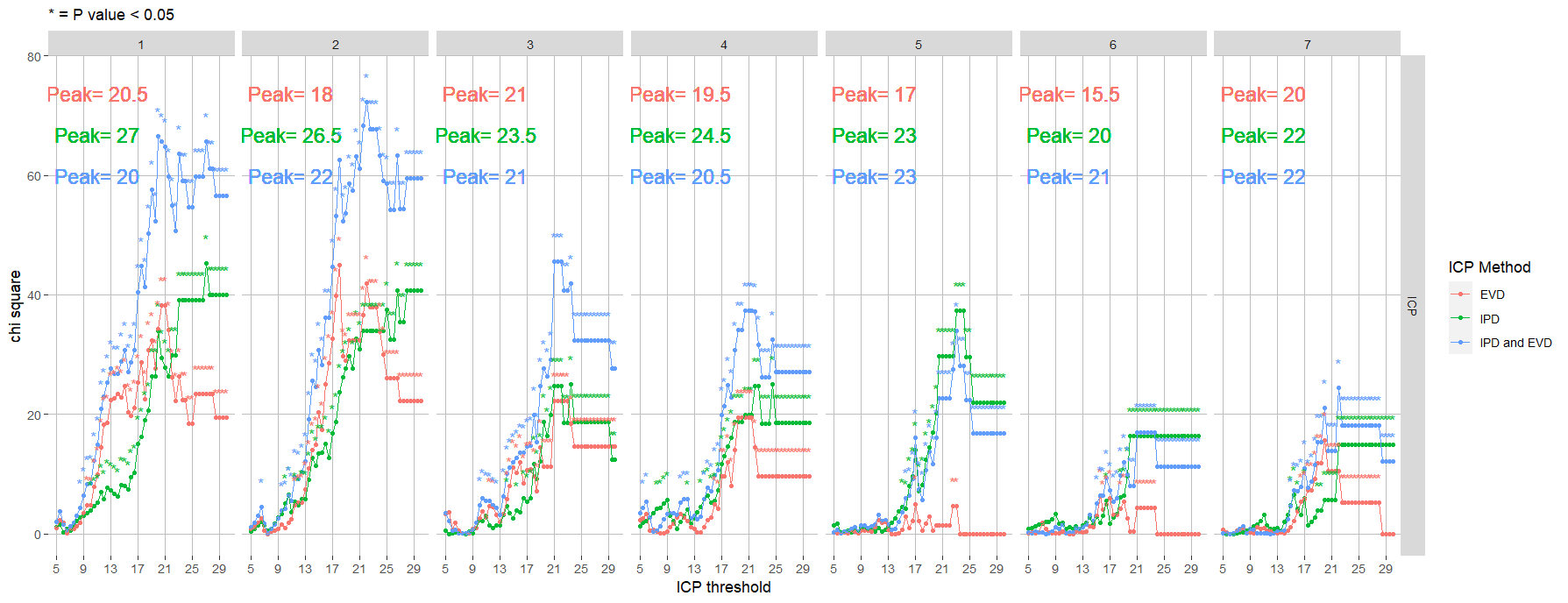


The figure displays a trend of a single day of care (day 1/2/3/4/5/6/7) and resulting ICP threshold. Each day is a single 24 hour period from the initial start of recording (ie. day 1 = 0-24 hours of recording, day 2 = 25-48 hours of recording, etc.). EVD, external ventricular drain; ICP, intracranial pressure; IPD, intraparenchymal monitoring;

# Appendix O. ICP for Each Day of Care: Favorable vs Unfavorable


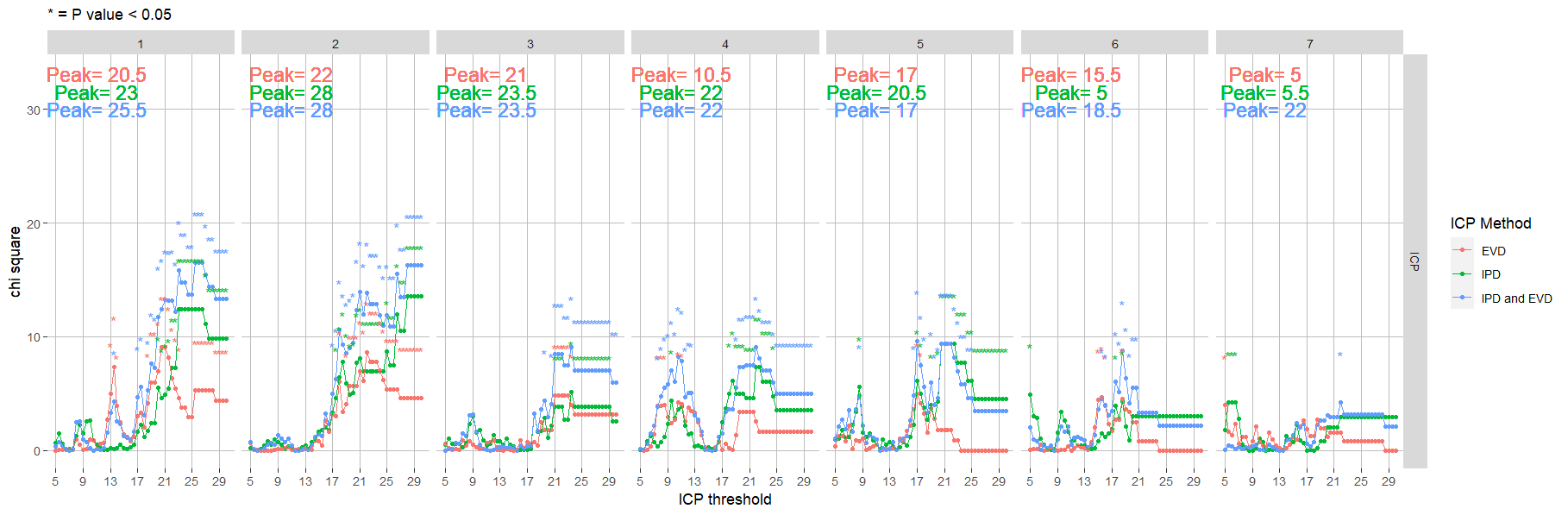


The figure displays a trend of a single day of care (day 1/2/3/4/5/6/7) and resulting ICP threshold. Each day is a single 24 hour period from the initial start of recording (ie. day 1 = 0-24 hours of recording, day 2 = 25-48 hours of recording, etc.). EVD, external ventricular drain; ICP, intracranial pressure; IPD, intraparenchymal monitoring;

# Appendix P. Non-Decompressive Patients


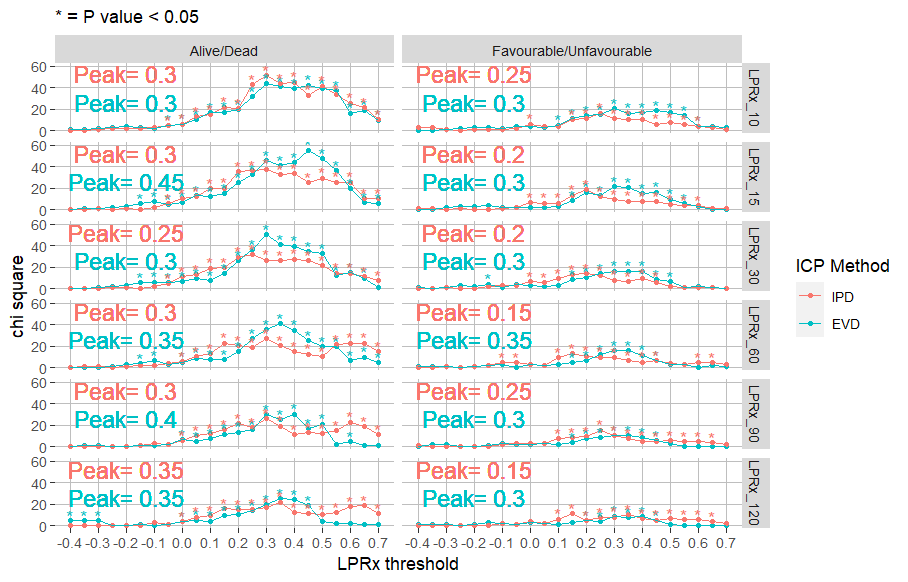

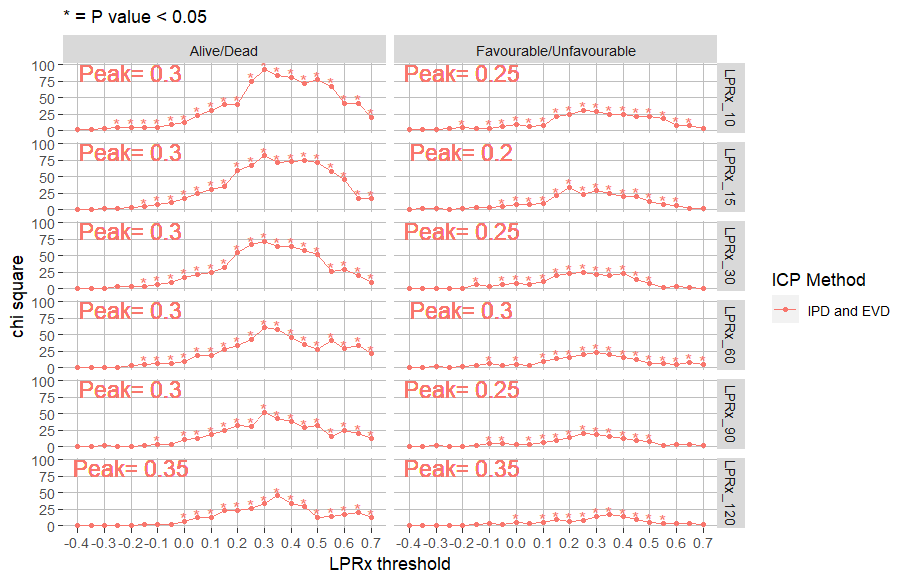


The figure displays a trend of a full data in patients without decompressive craniectomy. EVD, external ventricular drain; ICP, intracranial pressure; IPD, intraparenchymal monitoring.

# Appendix Q. % Time LPRx over Thresholds Results: Alive vs Dead


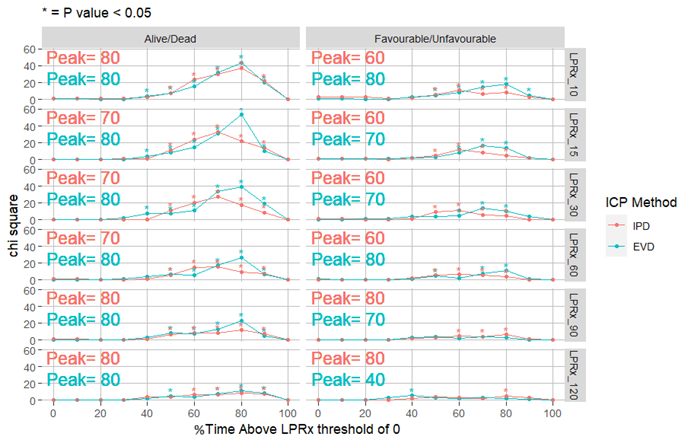

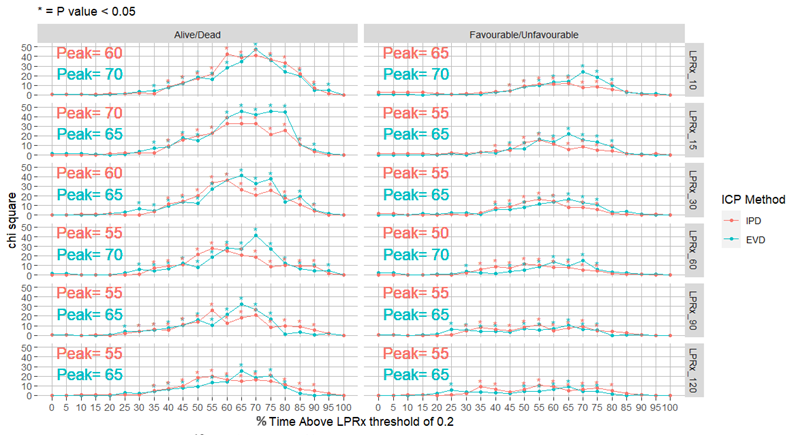

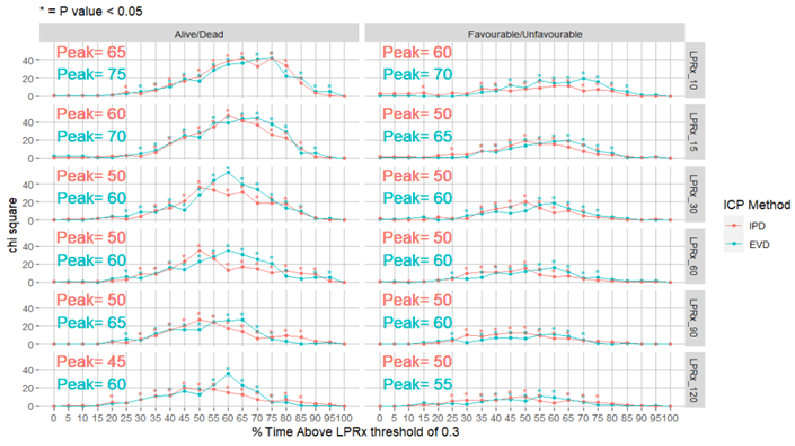


*The figures* display a trend of *different LPRx windows and the resulting % time over key thresholds (0, 0.2 and 0.3) with different ICP monitoring methods using minute by minute resolution. Similar overall results with IPD and EVD. Noting also that as threshold increase, % time decreases. EVD, external ventricular drain; ICP, intracranial pressure; IPD, intraparenchymal monitoring; LPRx, long pressure reactivity; _10, 10 minutes window; _15, 15 minutes; _30, 30 minutes; _60, 60 minutes; _90, 90 minutes; _120, 120 minutes.*

# Appendix R. First Days of Care and % Time LPRx > 0.3 Results: Alive vs Dead


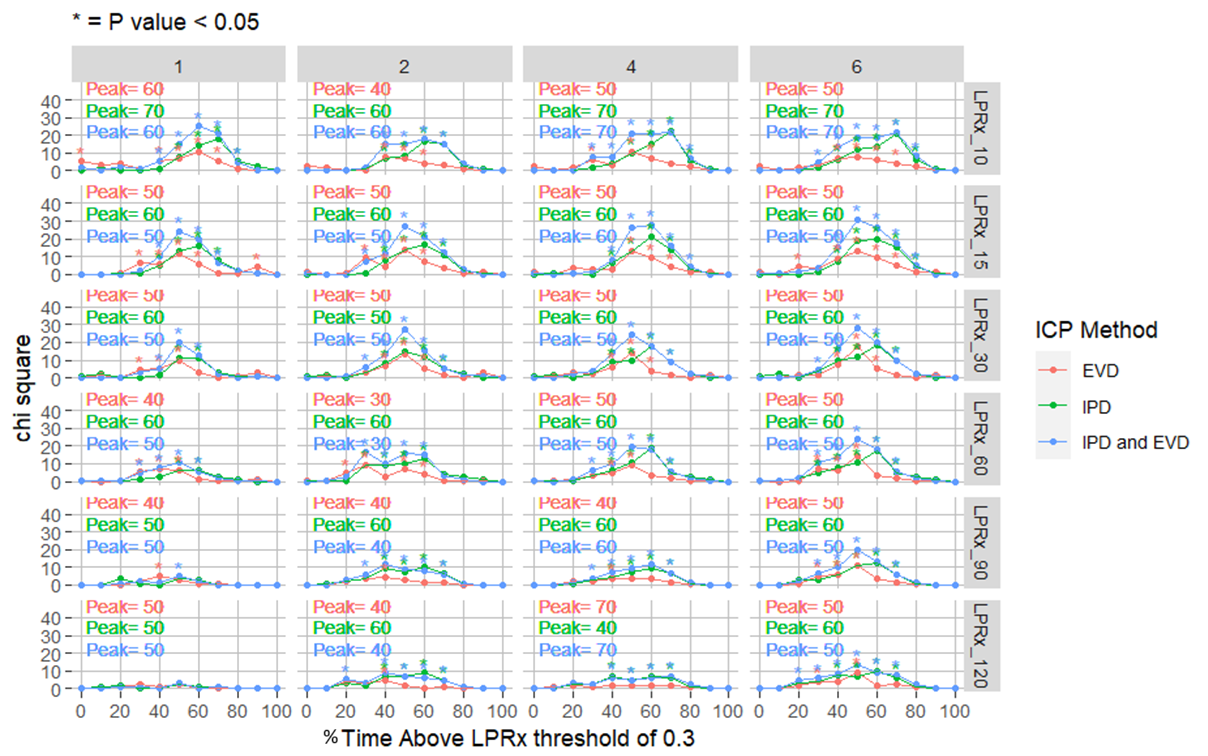


The figure displays a trend of the cumulative first days (1/2/4/6 days total) of care and the change in the resulting LPRx threshold using minute by minute resolution. EVD, external ventricular drain; ICP, intracranial pressure; IPD, intraparenchymal monitoring; LPRx, long pressure reactivity;

# Appendix S. First Days of Care and % Time LPRx > 0.3 Results: Favorable vs Unfavorable

­­­­­­
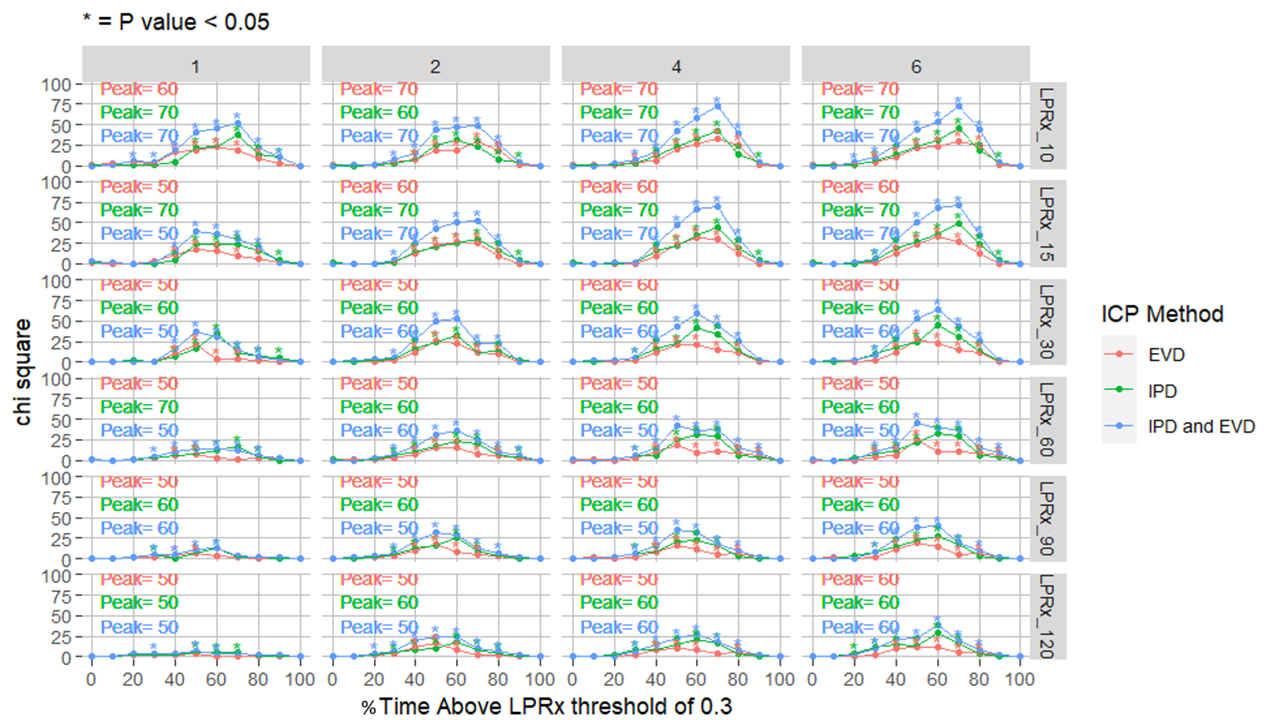


The figure displays a trend of the cumulative first days (1/2/4/6 days total) of care and the change in the resulting LPRx threshold using minute by minute resolution. EVD, external ventricular drain; ICP, intracranial pressure; IPD, intraparenchymal monitoring; LPRx, long pressure reactivity;

# Appendix T. % Time LPRx > 0.3 for Each Day of Care: Alive vs Dead


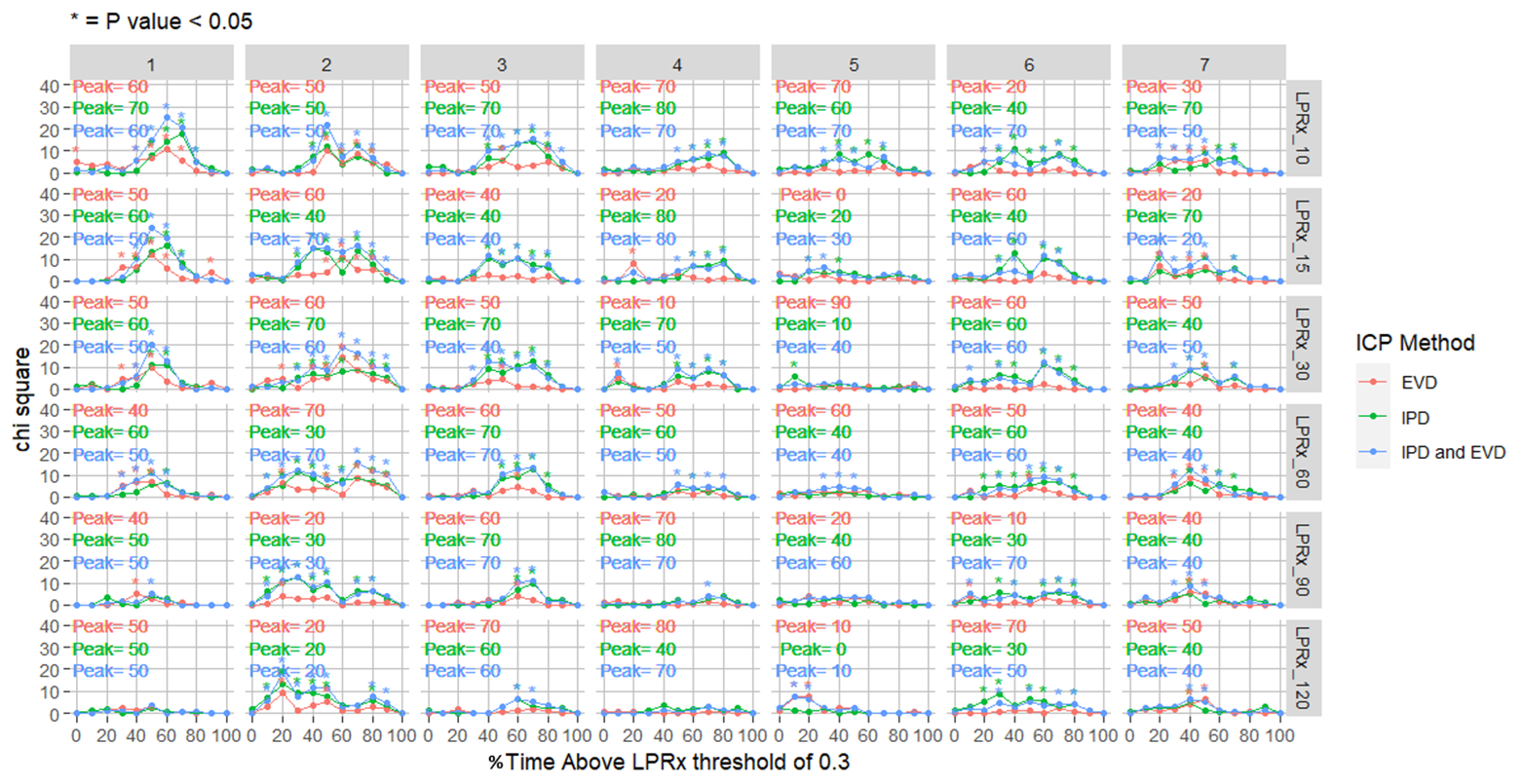


The figure displays a trend of a single day of care (day 1/2/3/4/5/6/7) and resulting LPRx threshold using minute by minute resolution. Each day is a single 24 hour period from the initial start of recording (ie. day 1 = 0-24 hours of recording, day 2 = 25-48 hours of recording, etc.). EVD, external ventricular drain; ICP, intracranial pressure; IPD, intraparenchymal monitoring; LPRx, long pressure reactivity;

# Appendix U. % Time LPRx > 0.3 for Each Day of Care: Favorable vs Unfavorable


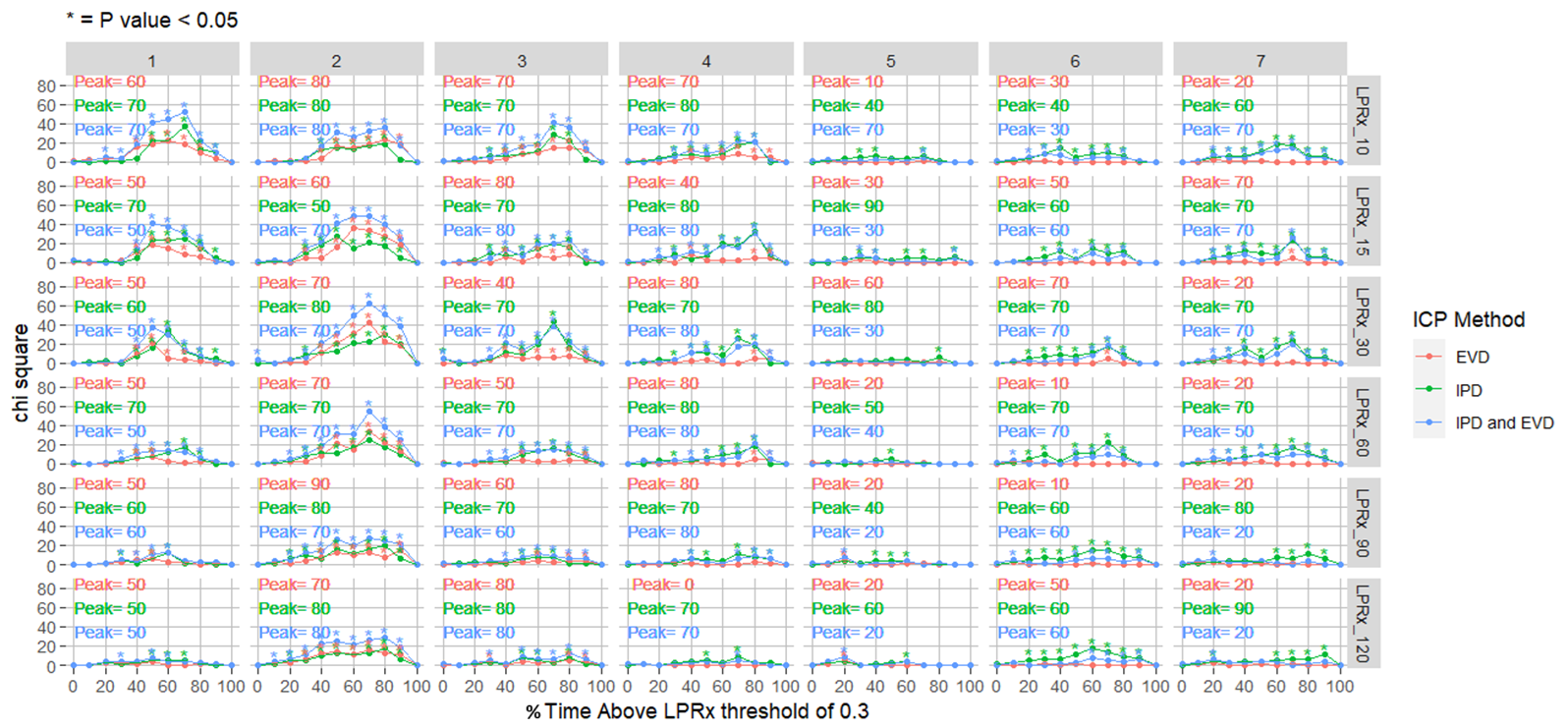


The figure displays a trend of a single day of care (day 1/2/3/4/5/6/7) and resulting LPRx threshold using minute by minute resolution. Each day is a single 24 hour period from the initial start of recording (ie. day 1 = 0-24 hours of recording, day 2 = 25-48 hours of recording, etc.). EVD, external ventricular drain; ICP, intracranial pressure; IPD, intraparenchymal monitoring; LPRx, long pressure reactivity;

# Appendix V. First Days of Care and % Time LPRx > 0 Results: Alive vs Dead


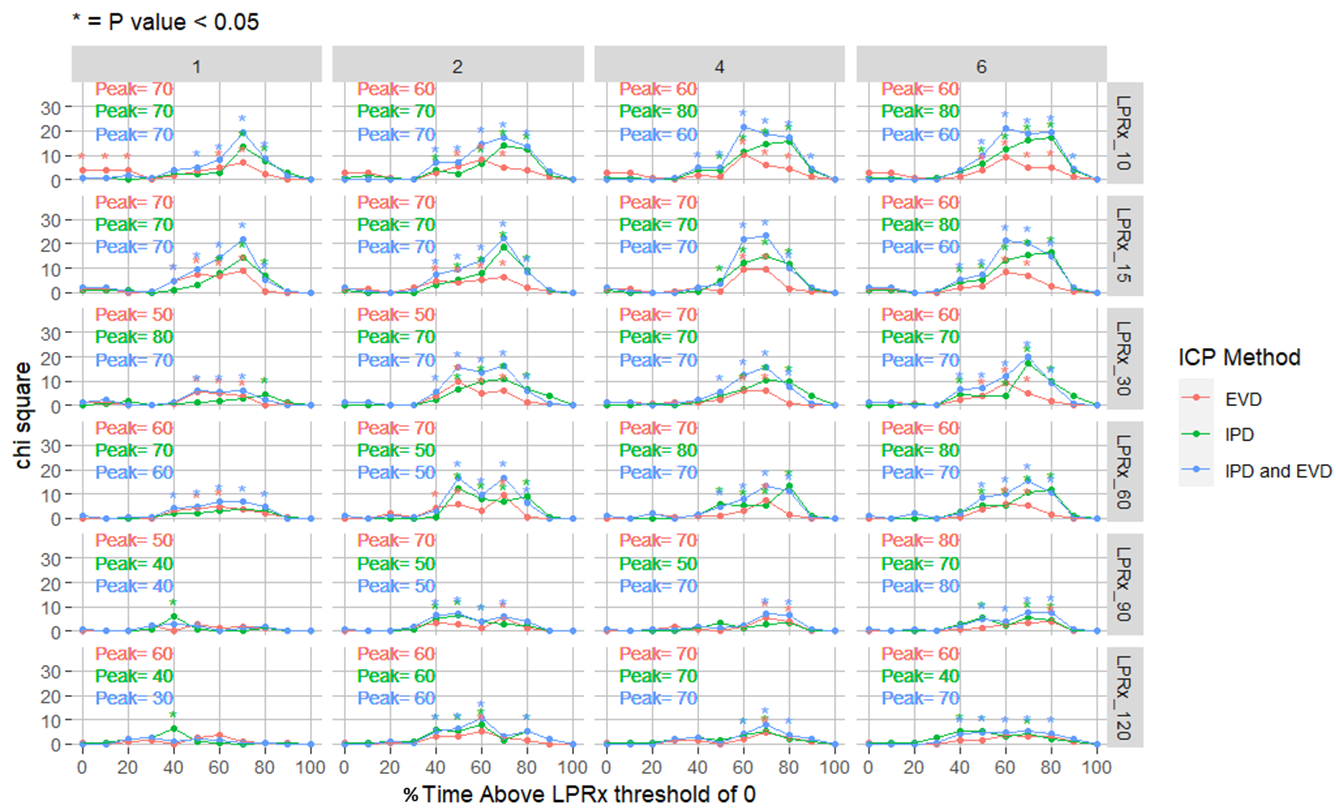


The figure displays a trend of the cumulative first days (1/2/4/6 days total) of care and the change in the resulting LPRx threshold using minute by minute resolution. EVD, external ventricular drain; ICP, intracranial pressure; IPD, intraparenchymal monitoring; LPRx, long pressure reactivity;

# Appendix W. First Days of Care and % Time LPRx > 0 Results: Favorable vs Unfavorable

­­­­­­
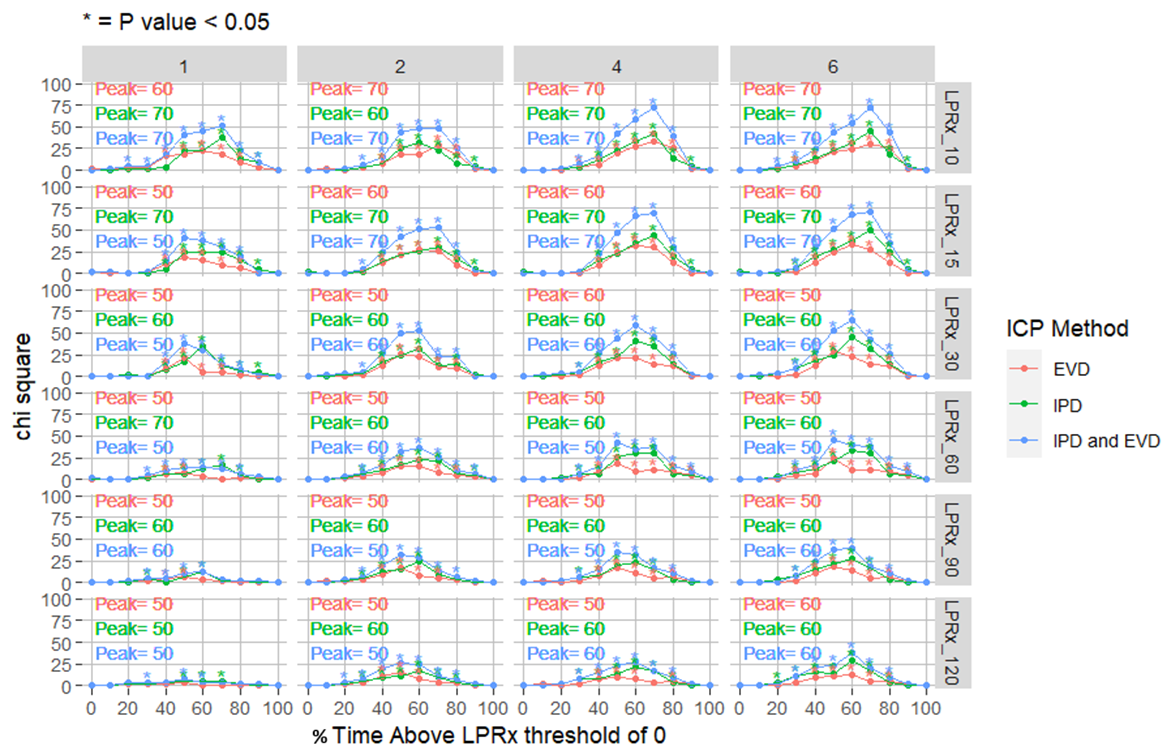


The figure displays a trend of the cumulative first days (1/2/4/6 days total) of care and the change in the resulting LPRx threshold using minute by minute resolution. EVD, external ventricular drain; ICP, intracranial pressure; IPD, intraparenchymal monitoring; LPRx, long pressure reactivity;

# Appendix X. % Time LPRx > 0 for Each Day of Care: Alive vs Dead


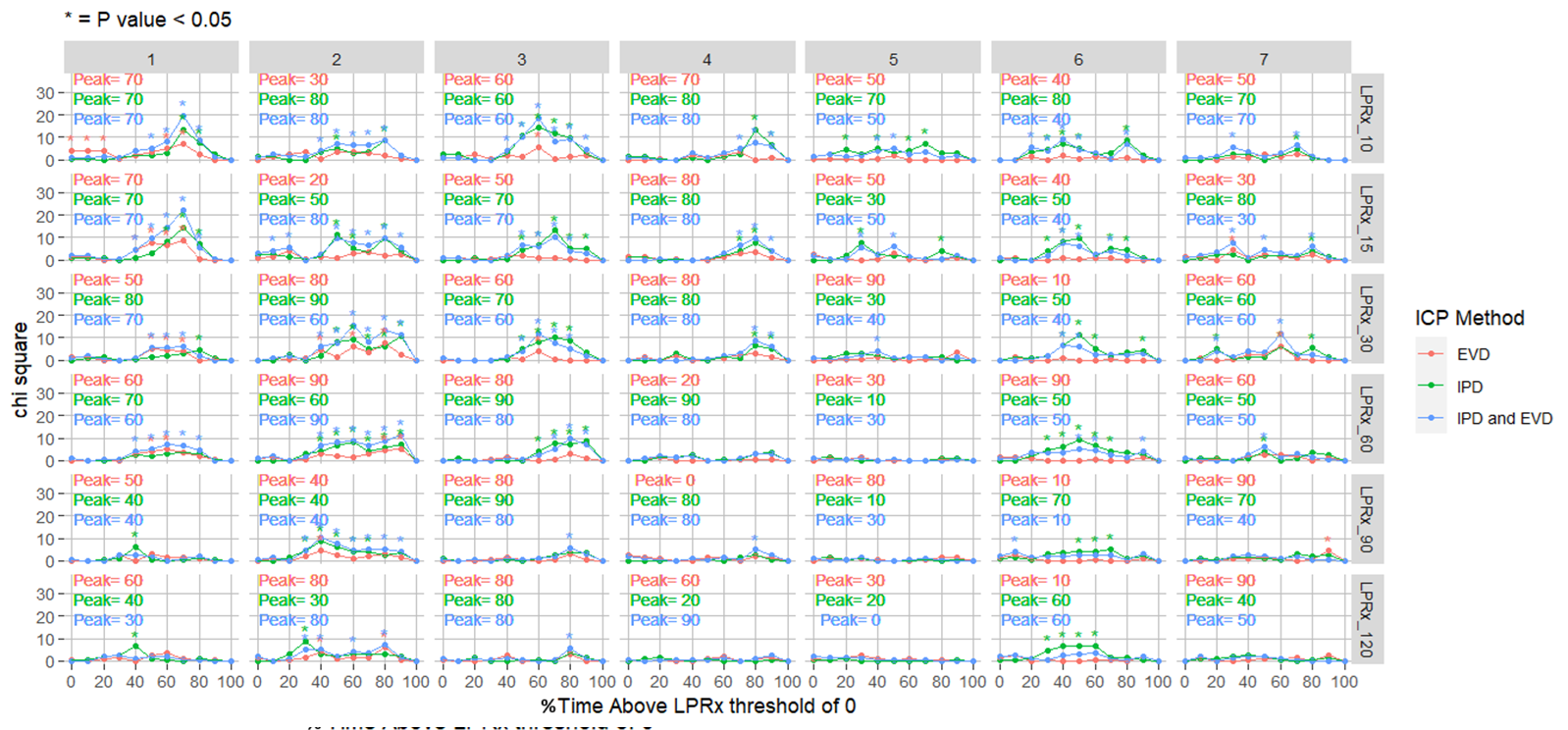


The figure displays a trend of a single day of care (day 1/2/3/4/5/6/7) and resulting LPRx threshold using minute by minute resolution. Each day is a single 24 hour period from the initial start of recording (ie. day 1 = 0-24 hours of recording, day 2 = 25-48 hours of recording, etc.). EVD, external ventricular drain; ICP, intracranial pressure; IPD, intraparenchymal monitoring; LPRx, long pressure reactivity;

# Appendix Y. % Time LPRx > 0 for Each Day of Care: Favorable vs Unfavorable


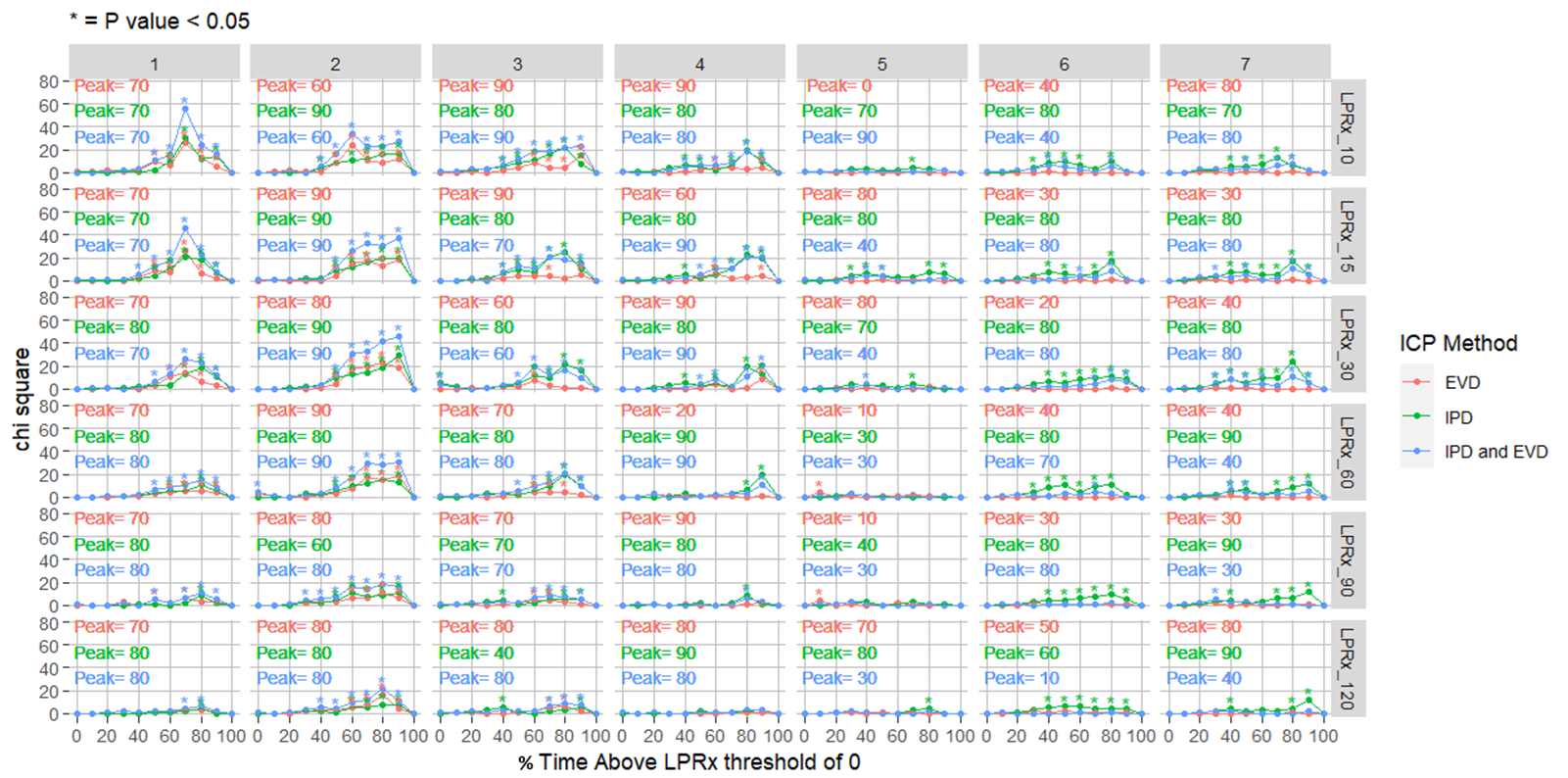


The figure displays a trend of a single day of care (day 1/2/3/4/5/6/7) and resulting LPRx threshold using minute by minute resolution. Each day is a single 24 hour period from the initial start of recording (ie. day 1 = 0-24 hours of recording, day 2 = 25-48 hours of recording, etc.). EVD, external ventricular drain; ICP, intracranial pressure; IPD, intraparenchymal monitoring; LPRx, long pressure reactivity;
